# Mendelian randomisation study of age at menarche and age at menopause and the risk of colorectal cancer

**DOI:** 10.1038/s41416-018-0108-8

**Published:** 2018-05-24

**Authors:** Sonja Neumeyer, Barbara L. Banbury, Volker Arndt, Sonja I. Berndt, Stephane Bezieau, Stephanie A. Bien, Dan D. Buchanan, Katja Butterbach, Bette J. Caan, Peter T. Campbell, Graham Casey, Andrew T. Chan, Stephen J. Chanock, James Y. Dai, Steven Gallinger, Edward L. Giovannucci, Graham G. Giles, William M. Grady, Jochen Hampe, Michael Hoffmeister, John L. Hopper, Li Hsu, Mark A. Jenkins, Amit Joshi, Susanna C. Larsson, Loic Le Marchand, Annika Lindblom, Victor Moreno, Mathieu Lemire, Li Li, Yi Lin, Kenneth Offit, Polly A. Newcomb, Paul D. Pharaoh, John D. Potter, Lihong Qi, Gad Rennert, Clemens Schafmayer, Robert E. Schoen, Martha L. Slattery, Mingyang Song, Cornelia M. Ulrich, Aung K. Win, Emily White, Alicja Wolk, Michael O. Woods, Anna H. Wu, Stephen B. Gruber, Hermann Brenner, Ulrike Peters, Jenny Chang-Claude

**Affiliations:** 1 Division of Cancer Epidemiology, German Cancer Research Center (DKFZ), 69120 Heidelberg, Germany; 2 Public Health Sciences Division, Fred Hutchinson Cancer Research Center, Seattle, WA 98124 USA; 3 Division of Clinical Epidemiology and Aging Research, German Cancer Research Center (DKFZ), 69120 Heidelberg, Germany; 4 Division of Cancer Epidemiology and Genetics, National Cancer Institute, National Institutes of Health, Bethesda, MD 20892-9776 USA; 5 Centre Hospitalier Universitaire (CHU), Nantes Service de Génétique Médicale, 44093 Nantes, France; 6 Colorectal Oncogenomics Group, Genetic Epidemiology Laboratory, Department of Pathology, The University of Melbourne, Melbourne, VIC 3010 Australia; 7 Centre for Epidemiology and Biostatistics, Melbourne School of Population and Global Health, The University of Melbourne, Parkville, VIC 3010 Australia; 8 Division of Research, Kaiser Permanente Medical Care Program of Northern California, Oakland, 94612 CA USA; 9 Epidemiology Research Program, American Cancer Society, Atlanta, GA 30329-4251 USA; 10 Center for Public Health Genomics, University of Virginia, Charlottesville, VA 22908 USA; 11 Clinical and Translational Epidemiology Unit and Division of Gastroenterology, Massachusetts General Hospital, Boston, MA 02115 USA; 12 Channing Division of Network Medicine, Brigham and Women’s Hospital and Harvard Medical School, Boston, MA 02115 USA; 13 Department of Medicine, Massachusetts General Hospital, Harvard Medical School, Boston, MA 02115 USA; 14 Division of Cancer Epidemiology and Genetics, National Cancer Institute, National Institutes of Health, Bethesda, MD 20892-9776 USA; 15 Lunenfeld-Tanenbaum Research Institute, Mount Sinai Hospital, Toronto, ON M5G 1 X 5 Canada; 16 Department of Epidemiology, Harvard School of Public Health, Boston, MA 02115 USA; 17 Department of Nutrition, Harvard School of Public Health, Boston, MA 02115 USA; 18 Department of Medicine, Harvard Medical School, Boston, MA 02115 USA; 19 Cancer Epidemiology & Intelligence Division, Cancer Council Victoria, Melbourne, VIC 3010 Australia; 20 Department of Medicine, Division of Gastroenterology, University of Washington School of Medicine, Seattle, WA 98195 USA; 21 Clinical Research Division, Fred Hutchinson Cancer Research Center, Seattle, WA 98109 USA; 22 Medical Department 1, University Hospital Dresden, TU Dresden, 01307 Dresden, Germany; 23 Institute of Environmental Medicine, Karolinska Institutet Solna, SE-171 77 Stockholm, Sweden; 24 Epidemiology Program, University of Hawaii Cancer Center, Honolulu, 96822 HI USA; 25 Department of Clinical Genetics, Karolinska University Hospital Solna, SE-171 77 Stockholm, Sweden; 26 Department of Molecular Medicine and Surgery, Karolinska Institutet Solna, SE-171 77 Stockholm, Sweden; 27 Catalan Institute of Oncology, Bellvitge Biomedical Research Institute (IDIBELL), 08028 Barcelona, Spain; 28 CIBER Epidemiología y Salud Pública (CIBERESP), 28029 Madrid, Spain and University of Barcelona, Barcelona, 08007 Spain; 29 Ontario Institute for Cancer Research, Toronto, ON M5G 0A3 Canada; 30 Department of Family Medicine and Community Health, Case Western Reserve University, Cleveland, OH 44106 USA; 31 Department of Medicine, Clinical Genetics Service, Memorial Sloan Kettering Cancer Center, New York, NY 10065 USA; 32 Centre for Cancer Genetic Epidemiology, Department of Public Health & Primary Care, University of Cambridge, Cambridge, CB2 8AR UK; 33 Department of Public Health Sciences, University of California, Davis, CA 95817 USA; 34 Bruce Rappaport Faculty of Medicine, Technion-Israel Institute of Technology, Haifa, Israel; 35 Clalit Health Services National Israeli Cancer Control Center, Haifa, 34361 Israel; 36 Department of Community Medicine and Epidemiology, Carmel Medical Center, Haifa, 34361 Israel; 37 Department of General and Thoracic Surgery, University Hospital Schleswig-Holstein, Campus Kiel, 24118 Kiel, Germany; 38 Department of Medicine and Epidemiology, University of Pittsburgh Medical Center, Pittsburgh, PA 15213 USA; 39 Department of Internal Medicine, University of Utah Health Sciences Center, Salt Lake City, UT 84108 USA; 40 Huntsman Cancer Institute and Department of Population Health Sciences, University of Utah, Salt Lake City, UT 84112 USA; 41 Genetic Medicine and Familial Cancer Centre, Royal Melbourne Hospital, Parkville, VIC 3050 Australia; 42 Department of Surgical Sciences, Uppsala University, Uppsala, SE-171 77 Sweden; 43 Faculty of Medicine, Memorial University of Newfoundland, St. John’s, NL A1C 5S7 Canada; 44 Department of Preventive Medicine, University of Southern California Keck School of Medicine, Los Angeles, CA 90033 USA; 45 Department of Medicine, University of Southern California Keck School of Medicine, Los Angeles, CA 90033 USA; 46 Division of Preventive Oncology, German Cancer Research Center (DKFZ) and National Center for Tumor Diseases (NCT), 69120 Heidelberg, Germany; 47 German Cancer Consortium (DKTK), German Cancer Research Center (DKFZ), 69120 Heidelberg, Germany; 48 Genetic Tumour Epidemiology Group, University Medical Center Hamburg-Eppendorf, University Cancer Center Hamburg, 20246 Hamburg, Germany

**Keywords:** Cancer epidemiology, Risk factors, Colorectal cancer, Cancer genetics, Cancer epidemiology

## Abstract

**Background:**

Substantial evidence supports an association between use of menopausal hormone therapy and decreased colorectal cancer (CRC) risk, indicating a role of exogenous sex hormones in CRC development. However, findings on endogenous oestrogen exposure and CRC are inconsistent.

**Methods:**

We used a Mendelian randomisation approach to test for a causal effect of age at menarche and age at menopause as surrogates for endogenous oestrogen exposure on CRC risk. Weighted genetic risk scores based on 358 single-nucleotide polymorphisms associated with age at menarche and 51 single-nucleotide polymorphisms associated with age at menopause were used to estimate the association with CRC risk using logistic regression in 12,944 women diagnosed with CRC and 10,741 women without CRC from three consortia. Sensitivity analyses were conducted to address pleiotropy and possible confounding by body mass index.

**Results:**

Genetic risk scores for age at menarche (odds ratio per year 0.98, 95% confidence interval: 0.95–1.02) and age at menopause (odds ratio 0.98, 95% confidence interval: 0.94–1.01) were not significantly associated with CRC risk. The sensitivity analyses yielded similar results.

**Conclusions:**

Our study does not support a causal relationship between genetic risk scores for age at menarche and age at menopause and CRC risk.

## Introduction

Colorectal cancer (CRC) is the third most common cancer worldwide and incidence rates are higher in men than in women.^1^ The sex-specific difference might be partly attributed to differential exposure to sex hormones, especially oestrogen.^2^ This hypothesis is partially supported by epidemiologic studies as well as a recent meta-analysis out of four randomised controlled trials, eight cohort and eight case–control studies, which have shown that use of exogenous sex hormones in the form of combined oestrogen–progestogen menopausal hormone therapy (MHT) is inversely associated with the risk of CRC.^3, 4^

Epidemiologic studies examining reproductive factors such as age at menarche and age at menopause with CRC risk have reported inconsistent results.^5–7^ A meta-analysis summarising evidence based on 22 studies published by Li et al.^8^ did not find a significant association between age at menarche and CRC risk. In a large prospective cohort of the NIH American Association of Retired Persons (AARP) Diet and Health Study with more than 214,000 postmenopausal women, an inverse association between age at menarche and CRC risk was observed for women without a history of MHT use, whereas increasing age at menopause was associated with higher CRC risk.^9^ It is possible that the inconsistency in results is due to recall bias or to improper adjustment for confounders, which are inherent limitations of observational studies. The Mendelian randomisation (MR) approach^10^ uses genetic variants as instrumental variables to test for the causal effect of an exposure risk factor on an outcome. Since the genetic variants in offspring are randomly distributed at conception independent of environmental factors given parental genotypes, confounding and reverse causation are less likely to occur in MR analyses. For genetic variants to function as valid instrumental variables in MR analyses, three assumptions have to be met: (1) the genetic variants have to be associated with the exposure risk factor; (2) the variants are not associated with any confounding variables of the exposure–outcome association; and (3) the variants are unrelated to the outcome except through the risk factor of interest.^11, 12^ Using variants associated with age at menarche and age at menopause instead of self-reported measure of the risk factors themselves, this approach can help to avoid issues of confounding, recall bias, and reverse causation. Evidence from MHT use suggests that more exposure to oestrogen reduces risk of CRC.^3, 4, 13^ Estrogen exerts its effects in colon cells predominantly through the nuclear receptor oestrogen-receptor β (ERβ),^14, 15^ which has mainly anti-proliferative effects^16^ and its expression is inversely related to cancer stage, tumour extent, and mortality.^17, 18^ Longer use of MHT appears to be associated with high ERβ expression in tumours.^19^ If similar mechanisms hold then longer endogenous oestrogen exposure (through earlier age at menarche and/or later age at menopause) would reduce CRC risk as well. To test this hypothesis, we conducted an MR analysis using summary data for single-nucleotide polymorphisms (SNPs) known to be associated with age at menarche and age at menopause from prior studies as well as using individual level data of three consortia.

## Materials and methods

### Study population

Epidemiological and genetic data were derived from 26 studies participating in three large consortia of CRC, the Genetics and Epidemiology of Colorectal Cancer Consortium (GECCO)^20^ (5386 cases and 5696 controls), the Colon Cancer Family Registry (CCFR)^21^ (1678 cases and 1188 controls) and the Colorectal Cancer Transdisciplinary (CORECT) Consortium^22^ (5880 cases and 3857 controls) (see Supplementary Tables 1 and 2). Different centres of CCFR participated as individual studies in GECCO and/or CORECT, and therefore were analysed as such. Any participant overlap between the three consortia was excluded. In total, 12,944 female colorectal cancer cases and 10,741 female controls, both of European ancestry were included. All participants provided written, informed consent and studies were approved by their respective institutional review boards. Women with incident invasive colorectal adenocarcinoma (International Classification of Disease Code, 9th revision (ICD-9), codes 153–154) were included as cases. Data on demographic factors and lifestyle were collected using in-person interviews or self-completed questionnaires. Data harmonisation was done centrally as previously described.^23^ Self-reported data for age at menarche and age at menopause have not been harmonised and therefore could not be used.

### Genotype data and imputation

Genotype information was available for all included studies. Details on genotyping, quality assurance, and imputation are included in the Supplementary Information. In short, SNPs were excluded based on call rate (<98% GECCO; <95% in CORECT), Hardy–Weinberg equilibrium in controls (*P* < 1 × 10^−4^), or low minor allele frequency (≤1%). Participants received a value of 0, 1, or 2 for carrying 0 (wild-type homozygous), 1 (heterozygous), or 2 (homozygous for the risk allele) alleles associated with higher age at menarche/age at menopause for each SNP. For imputed SNPs, participants were assigned continuous values between 0 and 2.

### Calculation of genetic risk scores

Two recent genome-wide association studies conducted by the REPROGEN consortium (Reproductive Genetics Consortium) involving up to ~370,000 women for the study on age at menarche and up to 69,360 women for the study on age at menopause identified 389 genetic variants associated with age at menarche^24^ and 54 SNPs associated with age at natural menopause^25^ at a genome-wide significance level (i.e., *P* < 5 × 10^−^^8^). Of the reported 389 genetic variants for age at menarche, 12 variants on sex chromosomes (which were not available in our datasets) were excluded. For further 42 missing variants, we used proxy SNPs in strong linkage disequilibrium (*R*² > 0.8, median *R*² = 0.985, *R*² range: 0.83–1.00) with effect alleles harmonised to reflect increase in age at menarche. Seven variants were excluded because no proxy was found. We checked for correlations between individual SNPs and excluded 12 correlated SNPs (*R*² > 0.01) (always the SNP with higher *P*-value for association with age at menarche was dropped). So data were available for 358 SNPs for this analysis (mean imputation quality score = 0.97) (Supplementary Table 5). For age at menopause, three correlated SNPs (*R*² > 0.01) were excluded. All remaining 51 SNPs for age at menopause were available in the datasets (mean imputation quality score = 0.98) (Supplementary Table 6). The genotype data described were used to construct genetic risk scores (GRS) as instrumental variables for age at menarche and age at menopause, respectively.

The GRS for the *k*th women is calculated by the sum of the number of risk increasing alleles carried (*G*) (imputed allele doses) for each SNP weighted by the reported beta-coefficient (*β*) for association with age at menarche and age at menopause.^24, 25^

Age at menarche:

$${\mathrm {GRS}}_K = \mathop {\sum}\limits_{n = 1}^{358} {{\it{\beta }}_{\it{n}}{\it{G}}_{{\it{Kn}}}}.$$

Age at menopause:

$${\mathrm {GRS}}_K = \mathop {\sum}\limits_{n = 1}^{51} {{\it{\beta }}_{{n}}{{G}}_{{{Kn}}}}.$$

As risk scores themselves do not have meaningful units, we scaled the risk scores in terms of age in years. In this way resulting odds ratios (OR) can be interpreted as the relative change in CRC risk per year older age at menarche/age at menopause. Scaling was done by dividing the GRS by regression coefficients of a linear regression of GRS on self-reported age at menarche (beta = 0.57) or age at menopause (beta = 0.98). These regression coefficients were obtained using the DACHS (Darmkrebs: Chancen der Verhütung durch Screening) study (853 control women) and the WHI (Womens’ Health Initiative) study (1492 control women) for which self-reported data on age at menarche were available and regression coefficients of the two studies were combined using meta-analysis.

An additional GRS as a surrogate for total lifetime exposure to endogenous oestrogen was calculated as the sum of the scaled risk scores for age at menarche and age at menopause.

$${\mathrm {GRS}}_{{\mathrm {time}}\,{\mathrm {period}}} = {\mathrm {GRS}}_{{\mathrm {age}}\,{\mathrm {at}}\, {{\mathrm {menarche}}}} + {\mathrm {GRS}}_{{\mathrm {age}}\,{\mathrm {at}}\,{\mathrm {menopause}}}.$$

For this analysis, risk scores for age at menarche were calculated as the sum of the number of alleles associated with decreasing age at menarche and the number of alleles associated with increasing age at menopause. So the GRS for time period of oestrogen exposure reflects higher risk for longer exposure to endogenous oestrogen. For this score, four SNPs (rs3136269, rs11031040, rs537244, rs4303811) were excluded due to high linkage disequilibrium (*R*² > 0.01) between age at menarche and age at menopause SNPs.

### Statistical analysis

#### Validating MR assumptions

The first assumption of MR regarding instrumental variable strength (i.e. association between the genetic variants and the exposure risk factor) was verified by calculating the *F*-statistic out of *F* = (*R*²(*n*−*K*−1))/((1−*R*²)*K*)), where *R*² refers to the variance explained by the instrumental variable,^24, 25^ *K* indicates the number of instrumental variables, and *n* stands for the sample size.^26^ An *F*-statistic >10 suggests that the genetic instrument is sufficiently strong.^27^ To evaluate the second assumption of MR (i.e. no association between genetic variants and potential confounders), we tested associations between GRS for age at menarche/age at menopause and the following risk factors for CRC: smoking status (ever/never), family history of CRC, education/educational level, ever aspirin/NSAID use (at least once per month for more than one year), body mass index (BMI) (continuous), MHT (oestrogen/progestin combined and oestrogen alone), using linear regression for continuous variables and logistic/multinomial logistic regression for categorical variables in a subset of the studies with available data (*n* = 6285 controls). To address the third assumption of MR, i.e. to assess the presence of pleiotropy, we applied the MR-Egger method.^28^ MR-Egger relies on the InSIDE assumption (Instrument Strength Independent of Direct Effect), which is the assumption that the pleiotropic effects of the genetic variants are not correlated with the effects of genetic variants on the risk factors. MR-Egger uses an inverse-variance weighted estimator and by plotting the SNP’s effect on the exposure against its effect on the outcome, the intercept term of MR-Egger provides a test for directional pleiotropy, i.e. the average effect of pleiotropy is non-zero, across all genetic variants used. If the average pleiotropic effect of all variants is zero and the InSide assumption is satisfied, pleiotropy is “balanced” and will not be detected. If the intercept differs from zero, it suggests horizontal pleiotropy, which means that some genetic variants affect the outcome through a pathway different from the exposure of interest. For visual inspection of pleiotropy, we used funnel plots of each SNPs ratio estimate against its precision (1/standard error of the ratio estimate).^29, 30^ Any deviation from symmetry would suggest pleiotropy.

### Estimation of causal effect

#### GRS based analyses

We examined the association between GRS and CRC risk using logistic regression models adjusted for study, age as well as principal components (PCs) of genetic ancestry (three PCs were used for GECCO and 10 PCs were used for CORECT) to account for potential population stratification. Summary results for GECCO and CORECT were derived using fixed-effects meta-analysis assuming that the included studies share a common effect size. As there are indications for differential associations of age at menarche with CRC risk according to menopausal hormone use,^9, 31^ stratified analyses by MHT were performed (only in GECCO where harmonised data were available). Additional stratified analyses were performed according to menopausal status (for age at menarche), combined menopausal oestrogen or progesterone therapy, oestrogen therapy alone, and BMI categories in kg/m² (BMI < 18.5: underweight, 18.5–24.9: normal weight, 25–30: overweight, >30: obese) (only for age at menopause). Due to reported differences in risk between colon and rectal cancer associated with hormone use,^32, 33^ we also conducted site-specific analyses for 4037 female colon cancer cases and 1184 rectal cancer cases in GECCO. Power calculations were conducted to estimate the magnitude of effects detectable with our study size assuming 5% alpha level and an *R*² of 0.069 for age at menarche and *R*² of 0.057 for age at menopause, which corresponds to the variance in age at menarche/age at menopause explained by the SNPs used for this analyses.^34^

It is known that BMI in childhood is strongly associated with age at menarche^35^ and that some SNPs associated with age at menarche could also have pleiotropic effects and therefore are related to BMI as well.^24^ To address this issue and thereby also account for violations of the third MR assumption, we conducted further BMI-specific sensitivity analyses. We adjusted for BMI in the logistic regression analysis using a weighted GRS for BMI comprising 77 SNPs previously reported to be associated with BMI at a genome-wide significance level in European subjects.^36^ For the second sensitivity analysis, we identified age at menarche SNPs showing pleiotropy by testing the association of these SNPs with BMI in a subset of our sample (*n* = 5832 cases/6285 controls) and found 29 SNPs to be associated with BMI at nominal significance (*P*-value <0.05). Two further SNPs overlapped with reported BMI-SNPs^36^ and 11 more SNPs were in high linkage disequilibrium (*R*² > 0.1) with BMI SNPs. A restricted GRS for age at menarche excluding the 42 BMI-associated SNPs (*n* = 316 SNPs) was constructed and then assessed for association with CRC risk. For the analysis of lifetime oestrogen exposure we also generated a restricted risk score excluding the same 42 BMI-associated SNPs.

#### Two-sample MR analyses

We performed two-sample MR analyses as sensitivity analyses using published summary statistics for SNP–exposure associations (age at menarche^24^/age at menopause^25^); SNP outcome associations were estimated in GECCO/CORECT (Supplementary Tables 8 and 9 for age at menarche/menopause-SNPs, respectively). We applied the weighted median estimator approach, which is robust against violations due to pleiotropic SNPs even when up to 50% of the genetic instruments are invalid.^37^ For this approach, we used SNP-exposure and SNP-outcome associations to build ratio estimates for each SNP. These estimates were ordered and weighted by the inverse of their variance. Bootstrapped standard errors were calculated and used for construction of 95% confidence intervals (CI). Furthermore, we assessed the slope of MR-Egger regression (see section on validation of MR assumptions) which yields a pleiotropy-adjusted estimate of the true causal effect.^28^

All analyses were conducted using R version 3.2.2 (R Foundation for Statistical Computing, Vienna, Austria). For supplementary figures, we used the R packages “Mendelian Randomisation”^38^ and “ggplot2”.

## Results

The assessment of the MR assumptions indicated that our instrumental variables for age at menarche and for age at menopause were both strong instruments (F-statistic for age at menarche = 1755, *R*² = 0.069^24^; *F*-statistic for age at menopause = 1431, *R*² = 0.057^25^. Secondly, we did not find significant associations between the GRS for age at menarche and CRC risk factors, including smoking, family history of cancer, education, aspirin/NSAID use, oestrogen/progestin therapy, oestrogen alone therapy, with the exception of BMI, which showed a significant association (Supplementary Table 3). Similarly, there was no association of the GRS for age at menopause with any of the tested risk factors (Supplementary Table 3). Table 1 shows the results of the MR analyses for age at menarche, age at menopause, and lifetime oestrogen exposure with CRC risk. Yearly increment in GRS for age at menarche was associated with CRC risk with an OR of 0.98 (95% CI: 0.95–1.02). Sensitivity analyses adjusting for BMI using a GRS yielded similar results for age at menarche (OR 0.99 per year, 95% CI: 0.95–1.02). Further sensitivity analysis using restricted risk scores for age at menarche (by excluding 42 BMI-associated SNPs) showed similar effect sizes for the association between age at menarche and CRC risk (OR 0.99 per year, 95% CI: 0.95–1.03). We also did not find evidence to support a causal association with risk of CRC for age at menopause (OR 0.98 per year, 95% CI: 0.94–1.01) or for lifetime oestrogen exposure (OR 0.99 per year, 95% CI: 0.97–1.02) using GRS-based analyses.

**Table 1:** Association between age at menarche/age at menopause and CRC risk using MR analyses and sensitivity analyses

		GRS-based analyses^b^						2-sample MR^f^			
		MR estimate^a^		Adjusted by GRS-BMI^a,c^		Restricted risk score^a,d^		MR-Egger^b,e^		Weighted median estimator^b^	
Variable (per year)	Cases/Controls	OR	95% CI	OR	95% CI	OR	95% CI	OR	95% CI	OR	95% CI
Age at menarche	12,944/10,741	0.98	0.95–1.02	0.99	0.95–1.02	0.99	0.95–1.03	0.99	0.83–1.17	1.00	0.90–1.11
Age at menopause	12,944/10,741	0.98	0.94–1.01	NA	NA	NA	NA	1.02	0.94–1.10	1.00	0.95–1.05
Time period of hormone exposure	12,944/10,741	0.99	0.97–1.02	0.99	0.97–1.02	0.99	0.97–1.01	0.97	0.93–1.01	1.00	0.95–1.05

*BMI* body mass index, *CCFR* Colon Cancer Family Registry, *CI* confidence interval, *CORECT* Colorectal Transdisciplinary study, *GECCO* Genetics and Epidemiology of Colorectal Cancer Consortium, *GRS* genetic risk score, *MR* Mendelian randomisation, *NA* not applicable, *OR* odds ratio per year, *SNPs* single-nucleotide polymorphisms, *se* standard error

^a^Logistic regression model adjusted for age, study, and principal components of genetic ancestry

^b^Meta-analysed estimate of GECCO/CORECT datasets (CCFR centres participated in GECCO or CORECT and were analysed as such)

^c^Additionally adjusted for a GRS for BMI out of 77 reported SNPs for BMI^36^

^d^42 BMI-associated SNPs were excluded from the age at menarche and time period of oestrogen exposure—risk scores

^e^Estimate derived from the slope of MR-Egger

^f^Estimates derived using summary statistics; se for calculation of CI obtained via bootstrapping

Results of the weighted median estimator approach also did not indicate causal association between age at menarche (OR per year 1.00, 95% CI: 0.90–1.11), age at menopause (OR per year 1.00, 95% CI: 0.95–1.05), or lifetime oestrogen exposure (OR per year 1.00, 95% CI: 0.95–1.05) with CRC risk.

The pleiotropy adjusted MR estimate (OR) derived from the slope of Egger regression was 0.99 (95% CI: 0.83–1.17) for age at menarche, 1.02 (95% CI: 0.94–1.10) for age at menopause, and 0.97 (95% CI: 0.93–1.01) for lifetime oestrogen exposure (details of results per GECCO and CORECT consortium in Supplementary Table 7). The intercept term from MR-Egger regression was centred at the origin for age at menarche (intercept term −0.0008, 95% CI: −0.007 to 0.005, *P-*value 0.80) and age at menopause (intercept term −0.009, 95% CI: −0.023 to 0.006, *P*-value 0.23), suggesting absence of strong directional pleiotropy (Supplementary Figures 1 and 2). Also the funnel plots for age at menarche and age at menopause appear to be generally symmetrical and therefore do not suggest presence of pleiotropy (Supplementary Figures 3 and 4). Table 2 shows stratified analyses for the association of GRS for age at menarche and CRC risk by menopausal status, combined oestrogen/progesterone therapy, and oestrogen alone therapy as well as cancer site based on GECCO data. None of these factors modified substantially the association between age at menarche and CRC risk. The site specific analysis showed no evidence for a difference in association between colon cancer (OR 0.97 per year, 95% CI: 0.91–1.02) and rectal cancer (OR 1.04 per year, 95% CI: 0.95–1.14). The analyses for age at menopause stratified by combined oestrogen/progesterone therapy, oestrogen alone therapy, BMI, and cancer site did not yield evidence for effect heterogeneity (Table 3). Power calculation shows that our study had >80 % power to detect an OR of 0.85 per standard deviation change in exposure variable (1.5 years for age at menarche, 4.8 years for age at menopause) but only around 50% power for an OR of 0.90 (Supplementary Table 4).

**Table 2:** Association of genetically predicted age at menarche with CRC risk according to subgroups, CCFR, GECCO Consortium

Subgroup	*N* (cases/controls)	OR^a^	95% CI	*P*-value	Heterogeneity *P-*value^b^
All	5832/6285	0.97	0.92–1.02	0.20	
**Menopausal status**					
Premenopausal	545/621	0.95	0.80–1.12	0.54	0.94
Postmenopausal	5263/5647	0.97	0.92–1.02	0.23	
**Menopausal hormone therapy combined**					
No	3266/3490	0.96	0.90–1.03	0.24	0.69
Yes	593/807	0.98	0.85–1.14	0.82	
**Oestrogen monotherapy**					
No	3120/3214	0.94	0.88–1.01	0.11	0.17
Yes	716/1088	1.04	0.91–1.19	0.55	
**Site**					
Colon	4037/6285	0.97	0.91–1.02	0.26	0.25^c^
Rectum	1184/6285	1.04	0.95–1.14	0.36	

*CCFR* Colon Cancer Family Registry, *CI* confidence interval, *GRS* genetic risk score, *OR* odds ratio per year

^a^All analyses adjusted for age, sex, study, and principal components of genetic ancestry

^b^*P*-value calculated using likelihood ratio tests comparing the model with and without interaction term

^c^*P-*value for heterogeneity was obtained in case-only analysis of colon vs. rectal cancer

**Table 3:** Association of genetically predicted age at menopause and CRC risk according to subgroups, CCFR, GECCO Consortium

	***N*** **(cases/controls)**	**OR** ^a^	95% CI	***P*** **-value**	**Heterogeneity** ***P-*** **value** ^b^
All	5832/6285	0.99	0.95–1.03	0.56	
**Combined oestrogen–progesterone therapy**					
No	3266/3490	0.99	0.93–1.05	0.78	0.75
Yes	593/807	0.96	0.85–1.10	0.58	
**Oestrogen monotherapy**					
No	3120/3214	0.99	0.93–1.05	0.27	0.76
Yes	716/1088	1.01	0.89–1.14	0.90	
**BMI**					
Normal weight	2146/2665	1.00	0.93–1.07	0.97	0.50
Overweight	1866/1918	0.99	0.91–1.07	0.77	
Obese	1284/1115	1.00	0.91–1.11	0.93	
Underweight	74/80	0.63	0.40–0.98	0.04	
**Site**					
Colon	4037/6285	0.98	0.94–1.03	0.53	0.61^c^
Rectum	1184/6285	0.98	0.91–1.06	0.65	

*BMI* body mass index, *CCFR* Colon Cancer Family Registry, *CI* confidence interval, *GRS* genetic risk score, *OR* odds ratio per year

^a^All analyses adjusted for age, sex, study, and principal components of genetic ancestry

^b^*P*-value calculated using likelihood ratio tests comparing the model with and without interaction term

^c^*P-*value for heterogeneity was obtained in case-only analysis of colon vs. rectal cancer

## Discussion

In this large MR study we aimed to clarify the inconsistent findings from observational studies regarding the association of age at menarche and age at menopause with CRC risk and thereby the role of endogenous oestrogen exposure. We investigated the association of GRS for age at menarche and age at menopause as surrogates for endogenous oestrogen exposure on CRC risk. Our results do not support an association between GRS for age at menarche and age at menopause and CRC risk, which under MR assumptions can be interpreted as absence of a causal effect.

In line with the findings of our study, several prospective studies and one meta-analysis reported no association^6, 8, 39^ between self-reported age at menarche and CRC risk, although some studies found an inverse association.^5, 9^ Two more recent prospective studies reported inverse associations with CRC risk for age at menarche only among never users of any MHT. In never users of hormone therapy, Zervoudakis et al.^9^ reported a hazard ratio of 0.73 (95% CI: 0.57–0.94) for age at menarche (>15 vs. 11–12 years) in association with risk of CRC and Murphy et al.^31^ found a hazard ratio of 0.72 (95% CI: 0.54–0.96) for age at menarche (>15 vs. 11–12 years). We therefore assessed the association of GRS for age at menarche with CRC risk stratified by ever/never use of MHT, separately for combined oestrogen–progesterone therapy and for oestrogen monotherapy. No difference in the association according to either combined oestrogen–progesterone therapy or oestrogen-alone therapy was found.

Higher BMI in childhood is associated with earlier age at menarche^24, 35^ and also with a higher risk for CRC.^40^ Therefore we conducted BMI-specific sensitivity analyses to account for violations of the MR assumptions by confounding and pleiotropy. Due to a strong association between childhood/adolescent BMI and adult BMI^41^ and also a high concordance between adolescent and adult BMI-SNPs,^42^ we accounted for adult BMI in sensitivity analyses. The effect sizes observed in the sensitivity analysis (by excluding BMI-associated SNPs and by adjustment using a GRS for BMI) were slightly smaller compared to results of the main analysis, which suggests that some of the effect was confounded by BMI. Our restricted risk-score might not be totally BMI-unrelated, considering that Day et al.^24^ found age at menarche variants that appear unrelated to BMI at a nominal level in their sample to be still BMI associated collectively (*P* = 4.2 × 10^−^^9^). Because of this strong interrelationship between age at menarche and BMI it is difficult to separate the SNPs into BMI-related and BMI-unrelated variants. So our adjustment of the analysis by GRS-BMI might more effectively control for BMI. Thus, any observed inverse relationship between age at menarche and CRC risk in previous observational studies could have been due to inadequate control of confounding by higher BMI in childhood.

For age at menopause, results of observational studies on the association with CRC risk have also been inconclusive. The large NIH-AARP study observed a statistically significant elevated risk for higher age at menopause in postmenopausal women (≥55 vs. <40; HR 1.50; 95% CI: 1.23–1.83),^9^ whereas most other studies reported null associations.^6, 31, 39^ Corresponding to the lack of association with age at menarche and age at menopause, the GRS for the reproductive period as indicator for the lifetime oestrogen exposure was also not significantly associated to CRC risk. Additional adjustment of that analysis by education, family history of CRC, ever regular aspirin use, MHT usage, BMI, and smoking did not substantially change the results (Supplementary Table 10). This is compatible with the observation of no association between the reproductive period (≥36 years vs. ≤30 years) and CRC risk in a prospective observational study conducted in Japan^43^. The GRS for lifetime for endogenous oestrogen exposure, which we constructed, does not account for other factors like parity or breast feeding, which influence overall oestrogen exposure; however, these factors have not been associated with CRC risk.

Results of prospective studies that investigated the association between serum levels of endogenous oestrogens and CRC risk have also been inconsistent. One study reported a positive association between circulating estradiol and CRC risk,^44^ another study observed an inverse relationship^45^ while most other studies found no associations.^46–48^ There are reports that earlier age at menarche is associated with higher oestrogen levels.^49, 50^ So oestrogen levels could also be a possible link between age at menarche and CRC risk. Due to the inconsistent results of these reports, further studies are needed to clarify these associations.

On the other hand, observational studies reported that exogenous oestrogen exposure by MHT mainly in the form of combined oestrogen–progestogen was associated with a reduced risk for CRC.^13, 51^ The Womens’s Health Initiative Clinical Trial (WHI-CT) reported no effect of oestrogen-alone therapy,^52, 53^ and a significant risk reduction for the association of oestrogen plus progestin vs. placebo and CRC risk,^33^ which was suggested to have resulted from diagnostic delay instead of true risk reduction^54^. However, a recent meta-analysis which summarised results of four clinical trials including WHI-CT and 16 observational studies concluded that there is consistent evidence to support a protective effect of MHT on CRC risk.^4^

Thus, it appears that exogenous and endogenous oestrogens, which also vary in absolute amount of oestrogen, may play different roles in the development of CRC, presumably by different mechanisms, which are not well understood. Oestrogen acts in colon cells predominantly through ERβ,^14, 15^ which exerts proapoptotic and anti-proliferative effects in the colon^16^ and its expression is reduced in tumour tissue.^17, 18^ According to an in vivo study, oestrogen treatment was associated with an increase in expression of ERβ in colon tissue,^55^ supporting a mechanism by which MHT may affect CRC risk. There is also some evidence that the protective effect of MHT on CRC risk may vary by the expression status of ERβ. Two studies found that the magnitude of risk reduction by MHT was different between colorectal tumours with higher and with lower expression of ERβ.^56, 57^ It is possible that the effect of endogenous oestrogens on CRC risk may be modulated by ERβ expression as well. Therefore, larger studies with data on expression of ERβ in colon tissue are warranted to assess whether the association of age at menarche/age at menopause and CRC risk differs by ERβ expression status.

In this MR study, we aimed to use proxies for start and endpoint of endogenous oestrogen exposure in women, specifically age at menarche/age at menopause, which themselves are complex traits influenced by many variants with only small effects on the trait. Although we did not see large pleiotropic effects using Egger regression, there might have been residual pleiotropy, which is difficult to exclude. Previous studies of age at menarche performed LD score regression using 123 SNPs associated with age at menarche and found, amongst others, genetic correlations with BMI, adult height, or type 2 diabetes. Residual pleiotropy related to adult height or type 2 diabetes, which have been reported to be associated with higher risk for CRC as well, cannot be fully excluded.^58^ In addition, Day et al. reported genetic correlations for the 54 age at menopause SNPs with adult obesity and other growth-related traits. The top menopause-SNPs were also associated with fasting glucose and were enriched in DNA repair pathways, yielding further sources of residual pleiotropy.^25^ Residual pleiotropy is a general limitation of MR, especially when exploring complex traits. When considering the recently published hypothesis of an omnigenic model of complex traits, coined “network pleiotropy”, essentially any regulatory variant in a trait-relevant cell-type can have some effect on the trait.^59^ This is because specific cell-types have specific regulatory networks, where any single variant could affect trait relevant genes, “core genes”, mediated through the same regulatory networks. So also for GWAS findings, it is highly likely that some genetic variants exhibit horizontal pleiotropy.^60^ As the selected age at menarche/age at menopause SNPs might also contribute tiny effects on further traits through network-pleiotropy, we cannot fully rule out pleiotropy. These limitations should be kept in mind and methods to explore the impact of such effects should be developed. That said, our sensitivity analyses especially Egger regression, did not indicate large pleiotropic effects.

The second assumption of MR is that the IV is not associated with confounding factors of the observational association between age at menarche/menopause and CRC risk. We were able to exclude most risk factors for CRC (smoking, family history of CRC, education, aspirin use, MHT) as confounding variables except for BMI, which was accounted for using several sensitivity analyses. In addition, substantial overlap between datasets used for estimating SNP-exposure and SNP-outcome associations would bias results in the direction of the observational estimate. There was some overlap between the studies (see Supplementary Information on participant overlap) but unlikely to have substantially influenced the results.

Strengths of our study include the large sample size, the availability of centrally harmonised data, and the robustness of the instrumental variables. Power calculations showed that our study has limited power to detect weak effects. Therefore, we cannot exclude a weak association of CRC with age at menarche or age at menopause. In summary, in our large MR study, evidence is limited for causal associations between age at menarche/age at menopause and CRC risk.

## Electronic supplementary material

Supplementary Information

Supplementary Table 8

Supplementary Table 9

## Availability of data and materials

Genotyping data of the GECCO studies will soon be available at the database of Genotypes and Phenotypes (dbGaP) for download at the accession number: phs001078.v1.p1.
