# Supplementary material for: Mendelian randomisation study of age at menarche and age at menopause and the risk of colorectal cancer
Source: Br J Cancer. 2018 May 24;118(12):1639–47. doi: 10.1038/s41416-018-0108-8 (PMC6008474; doi:10.1038/s41416-018-0108-8)
Supplement: Supplementary file 1 — Supplementary Information [file 41416_2018_108_MOESM1_ESM.docx]

# Mendelian randomization study of age at menarche and age at menopause and the risk of colorectal cancer

**Authors and affiliations:**

Sonja Neumeyer^1^, Barbara L. Banbury², Volker Arndt³, Sonja I. Berndt^4^, Stephane Bezieau^5^, Stephanie A. Bien^2^, Dan D Buchanan^6,7^, Katja Butterbach^1^, Bette J. Caan^8^, Peter T. Campbell^9^, Graham Casey^10^, Andrew T. Chan^11,12,13^, Stephen J. Chanock^14^, James Y Dai^2^, Steven Gallinger^15^, Edward L. Giovannucci^12,16,17,18^, Graham G. Giles^7,19^, William M. Grady^20,21^, Jochen Hampe^22^, Michael Hoffmeister^3^, John L. Hopper^7^, Li Hsu², Mark A. Jenkins^7^, Amit Joshi^13,16^, Susanna C. Larsson^23^, Loic Le Marchand^24^, Annika Lindblom^25,26^, Victor Moreno^27,28^, Mathieu Lemire^29^, Li Li^30^, Yi Lin², Kenneth Offit^31^, Polly A. Newcomb², Paul D. Pharaoh^32^, John D. Potter², Lihong Qi^33^, Gad Rennert^34,35,36^, Clemens Schafmayer^37^, Robert E. Schoen^38^, Martha L. Slattery^39^, Mingyang Song^11,13,17^, Cornelia M. Ulrich^40^, Aung K. Win^7,41^, Emily White², Alicja Wolk^23,42^, Michael O. Woods^43^, Anna H. Wu^44^, Stephen B. Gruber^45^, Hermann Brenner^3,46,47^, Ulrike Peters², Jenny Chang-Claude^1,48^

^1^ Division of Cancer Epidemiology, German Cancer Research Center (DKFZ), Heidelberg, Germany; ²Public Health Sciences Division, Fred Hutchinson Cancer Research Center, Seattle, WA, USA; ³Division of Clinical Epidemiology and Aging Research, German Cancer Research Center (DKFZ), Heidelberg, Germany. ^4^Division of Cancer Epidemiology and Genetics, National Cancer Institute, National Institutes of Health, Bethesda, MD, USA; ^5^Centre Hospitalier Universitaire (CHU) Nantes, Service de Génétique Médicale, Nantes, France; ^6^Colorectal Oncogenomics Group, Genetic Epidemiology Laboratory, Department of Pathology, The University of Melbourne, Victoria, Australia; ^7^Centre for Epidemiology and Biostatistics, Melbourne School of Population and Global Health, The University of Melbourne, Parkville, Victoria, Australia; ^8^Division of Research, Kaiser Permanente Medical Care Program of Northern California, Oakland, USA; ^9^Epidemiology Research Program, American Cancer Society, Atlanta, GA, USA;^10^Center for Public Health Genomics, University of Virginia, Charlottesville, VA, USA; ^11^ Clinical and Translational Epidemiology Unit and Division of Gastroenterology, Massachusetts General Hospital, Boston, MA, USA; ^12^Channing Division of Network Medicine, Brigham and Women's Hospital and Harvard Medical School, Boston, MA, USA.; ^13^Department of Medicine, Massachusetts General Hospital, Harvard Medical School, Boston, MA, USA, ^14^ Division of Cancer Epidemiology and Genetics, National Cancer Institute, National Institutes of Health, Bethesda, MD, USA; ^15^ Lunenfeld-Tanenbaum Research Institute, Mount Sinai Hospital, Toronto, Ontario, Canada; ^16^ Department of Epidemiology, Harvard School of Public Health, Boston, MA, USA; ^17^ Department of Nutrition, Harvard School of Public Health, Boston, MA, USA; ^18^Department of Medicine, Harvard Medical School, Boston, MA, USA; ^19^ Cancer Epidemiology & Intelligence Division, Cancer Council Victoria, Melbourne, Victoria, Australia.; ^20^Department of Medicine, Division of Gastroenterology, University of Washington School of Medicine, Seattle, Washington, USA., ^21^Clinical Research Division, Fred Hutchinson Cancer Research Center, Seattle, WA, USA ^22^Medical Department 1, University Hospital Dresden, TU Dresden, Dresden, Germany., ^23^Institute of Environmental Medicine, Karolinska Institutet Solna, Stockholm, Sweden, ^24^Epidemiology Program, University of Hawaii Cancer Center, Honolulu, HI, USA;^25^Department of Clinical Genetics, Karolinska University Hospital Solna, Stockholm, Sweden. ^26^ Department of Molecular Medicine and Surgery, Karolinska Institutet Solna, Stockholm, Sweden, ^27^Catalan Institute of Oncology, Bellvitge Biomedical Research Institute (IDIBELL), Barcelona, Spain; ^28^CIBER Epidemiología y Salud Pública (CIBERESP), Madrid, Spain; University of Barcelona, Barcelona, Spain. ^29^ Ontario Institute for Cancer Research, Toronto, Canada; ^30^Department of Family Medicine and Community Health, Case Western Reserve University, Cleveland, Ohio, USA., ^31^ Department of Medicine, Clinical Genetics Service, Memorial Sloan Kettering Cancer Center, New York, New York, USA., ^32^Centre for Cancer Genetic Epidemiology, Department of Public Health & Primary Care, University of Cambridge, Cambridge, UK, ^33^Department of Public Health Sciences, University of California, Davis, CA, USA; ^34^Bruce Rappaport Faculty of Medicine, Technion-Israel Institute of Technology, Haifa, Israel. ^35^Clalit Health Services National Israeli Cancer Control Center, Haifa, Israel.;  ^36^Department of Community Medicine and Epidemiology, Carmel Medical Center, Haifa, Israel, ^37^Department of General and Thoracic Surgery, University Hospital Schleswig-Holstein, Campus Kiel, Kiel, ^38^Department of Medicine and Epidemiology, University of Pittsburgh Medical Center, Pittsburgh, PA, USA; ^39^Department of Internal Medicine, University of Utah Health Sciences Center, Salt Lake City, UT, USA; ^40^Huntsman Cancer Institute and Department of Population Health Sciences, University of Utah, Salt Lake City, Utah, USA, ^41^Genetic Medicine and Familial Cancer Centre, Royal Melbourne Hospital, Parkville, Victoria, Australia; ^42^Department of Surgical Sciences, Uppsala University, Uppsala, Sweden, ^43^Faculty of Medicine, Memorial University of Newfoundland, St. John's, Newfoundland, Canada, ^44^Department of Preventive Medicine, University of Southern California Keck School of Medicine, Los Angeles, California; ^45^Department of Medicine, Keck School of Medicine, University of Southern California, Los Angeles, CA, USA, ^46^Division of Preventive Oncology, German Cancer Research Center (DKFZ) and National Center for Tumor Diseases (NCT), Heidelberg, Germany; ^47^German Cancer Consortium (DKTK), German Cancer Research Center (DKFZ), Heidelberg, Germany; ^48^Genetic Tumour Epidemiology Group, University Medical Center Hamburg-Eppendorf, University Cancer Center Hamburg, Hamburg, Germany.

**Correspondence:**

Prof. Dr. Jenny Chang-Claude,

Division of Cancer Epidemiology,

German Cancer Research Center (DKFZ),

Im Neuenheimer Feld 581, Heidelberg, 69120, Germany.

E-mail: [j.chang-claude@dkfz-heidelberg.de](mailto:j.chang-claude@dkfz-heidelberg.de)

Supplementary Table 1. Description of characteristics by study – GECCO

|  |  | **Controls** | | | | **Cases** | | |
| --- | --- | --- | --- | --- | --- | --- | --- | --- |
|  | **Study Design** | **n** | **Age (mean (SD))** | | **BMI (mean (SD))** | **n** | **Age (mean (SD))** | **BMI (mean (SD))** |
| French Association STudy Evaluation RISK for sporadic colorectal cancer (ASTERISK) | Case-Control | 423 | | 62.7 (10.5) | NA | 340 | 69.3 (10.8) | NA |
| Colon Cancer Family Registry (CCFR) | Case-Control | 589 | | 56.9 (11.4) | 26.4 (6.1) | 446 | 52.1 (11.6) | 26.8 (6.1) |
| Colorectal Cancer Studies 2 & 3 (Colo2&3) | Case-Control | 54 | | 65.7 (10.6) | 24.9 (5.7) | 40 | 66.3 (12.0) | 24.4 (5.3) |
| Darmkrebs: Chancen der Verhütung durch Screening (DACHS) | Case-Control | 860 | | 69.4 (10.1) | 25.5 (4.3) | 968 | 69.3 (11.4) | 26.5 (4.6) |
| Diet, Activity and Lifestyle Study (DALS) | Case-Control | 529 | | 64.3 (9.8) | 26.2 (5.1) | 493 | 64.2 (10.2) | 27.1 (5.8) |
| Multiethnic Cohort Study (MEC) | Cohort | 163 | | 63.3 (7.7) | 25.7 (4.7) | 148 | 63.4 (7.5) | 26.6 (5.9) |
| Nurses’ Health Study (NHS) | Cohort | 1343 | | 58.8 (6.8) | 25.2 (4.4) | 806 | 57.9 (6.8) | 25.6 (4.5) |
| Ontario Familial Colorectal Cancer Registry (OFCCR) | Case-Control | 225 | | 62.3 (8.1) | 25.6 (4.8) | 348 | 62.2 (7.7) | 25.7 (4.5) |
| Prostate, Lung, Colorectal, and Ovarian Cancer Screening Trial (PLCO) | Cohort | 327 | | 63.9 (5.2) | 26.5 (5.0) | 415 | 64.2 (5.3) | 27.5 (5.5) |
| Postmenopausal Hormone Study (PMH) | Case-Control | 116 | | 61.3 (7.4) | 25.5 (4.8) | 271 | 63.2 (6.8) | 27.8 (6.2) |
| VITamins And Lifestyle (VITAL) | Cohort | 130 | | 67.5 (5.8) | 26.4 (4.9) | 124 | 66.8 (5.8) | 28.0 (6.4) |
| Women’s Health Initiative (WHI) | Cohort | 1526 | | 66.4 (6.5) | 27.6 (5.5) | 1433 | 66.4 (6.7) | 28.3 (5.7) |

Abbreviations: BMI, body mass index; SD, standard deviation.

Supplementary Table 2. Description of characteristics by study - CORECT

|  |  | **Controls** | |  | **Cases** | | |
| --- | --- | --- | --- | --- | --- | --- | --- |
|  | **Study Design** | **n** | **Age (mean (SD))** |  | | **n** | **Age (mean (SD))** |
| Colon-Cancer Family Registry (CCFR) | Case-Control | 599 | 51.6 (12.4) |  | | 1232 | 53.2 (12.2) |
| ColoCare Heidelberg | Case-Series | 19 | 53.0 (10.1) |  | | 63 | 63.4 (10.8) |
| ColoCare Seattle | Case-Series | NA | NA |  | | 72 | 55.8 (12.3) |
| The American Cancer Society’s Cancer Prevention Study-II Nutrition Cohort (CPSII) | Case-Control | 260 | 68.2 (5.6) |  | | 268 | 68.7 (5.6) |
| ESTHER / VERDI | Case-Control | 147 | 65.3 (7.1) |  | | 136 | 65.5 (8.4) |
| Kentucky case–control study | Case-Control | 573 | 66.7 (6.6) |  | | 524 | 61.5 (10.4) |
| Kiel | Case-Control | NA | NA |  | | 480 | 62.4 (9.8) |
| Melbourne Collaborative Cohort Study (MCCS) | Cohort | 308 | 60.3 (7.3) |  | | 340 | 59.9 (7.9) |
| Multiethnic Cohort Study (MEC) | Cohort | 33 | 62.8 (8.4) |  | | 34 | 59.7 (8.6) |
| Molecular Epidemiology of Colorectal Cancer Study (MECC) | Case-Control | 54 | 67.8 (12.2) |  | | 105 | 66.2 (12.2) |
| The Memorial Sloan Kettering cohort (MSKCC) | Case-Control | NA | NA |  | | 5 | 57 (12.8) |
| Newfoundland Familial Colorectal Cancer Registry case–control study (NFCCR) | Case-Control | 197 | 58.2 (8.5) |  | | 73 | 60.1 (8.7) |
| Nurses' Health Study 2 (NHS2) | Cohort | 78 | 37.2 (4.1) |  | | 85 | 37.0 (4.5) |
| Spain | Case-Control | 366 | 62.9 (12.6) |  | | 269 | 66.5 (11.9) |
| The Swedish Low-Risk Colorectal Cancer Study | Cohort | 1083 | 61.7 (0) |  | | 1131 | 61.7 (0) |
| Swedish Mammography Cohort (SMC) | Cohort | 348 | 63.7 (8.2) |  | | 234 | 64.6 (9.1) |
| SEARCH (Studies of Epidemiology and Risk Factors in Cancer Heredity) | Case-Control | 46 | 53.6 (7.3) |  | | 1778 | 62.8 (8.2) |
| USC-HRT-CRC | Case-Control | 345 | 64.8 (6.8) |  | | 283 | 66.6 (5.4) |

Abbreviations: SD, standard deviation; NA, not applicable.

Supplementary Table 3: Association between confounders and the polygenic risk scores for age at menarche and age at menopause among controls in GECCO

|  | **Genetic risk score–age at menarche (scaled per year)^a^** | **Genetic risk score–age at menopause (scaled per year)^a^** |
| --- | --- | --- |
| Ever Smoking, OR (95% CI) | 0.97 (0.90, 1.04) | 1.03 (0.97, 1.09) |
| BMI (kg/m²), β (95% CI) | -0.08 (-0.11, -0.04) | -0.01 (-0.04, 0.02) |
| Education, OR (95% CI) |  |  |
| Less than high school graduate | Reference | Reference |
| High school graduate | 1.04 (0.90, 1.20) | 1.01 (0.89, 1.14) |
| Some college or graduate school | 1.01 (0.87, 1.17) | 1.00 (0.88, 1.14) |
| Graduate degree | 1.06 (0.91, 1.23) | 0.99 (0.87, 1.13) |
| Family history of CRC, OR (95% CI) | 1.01 (0.92, 1.12) | 1.05 (0.96, 1.15) |
| Aspirin/NSAIDS use, OR (95% CI) | 0.98 (0.91, 1.06) | 1.02 (0.95, 1.09) |
| Menopausal hormone therapy (Estrogen/Progestin combined), OR (95% CI) | 1.04 (0.94, 1.16) | 1.00 (0.91, 1.11) |
| Menopausal hormone therapy (estrogen-alone), OR (95% CI) | 0.97 (0.88, 1.07) | 1.07 (0.98, 1.17) |

Abbreviations: BMI, body mass index; CI, confidence interval; NSAIDs, no-steroidal anti-inflammatory drugs; OR, odds ratio.
^a^all analyses adjusted by age, study and three principal components of genetic ancestry.

**Supplementary Table 4**. Statistical power of the study for detecting different odds ratios at 5% type I error

| **Cancer Type** | | **N (cases/ controls)** | **Proportion of controls per 1 case** | | **OR** ^a^  **0.80** | | | **OR** ^a^  **0.85** | | **OR** ^a^  **0.90** | **OR** ^a^  **0.95** | **OR per year**  **(95% CI) DACHS** ^b^  (937 cases/  762 controls) | **OR per SD (95% CI) DACHS** ^b,c^ | **OR per year (95% CI)**  **WHI**^b^  (1393 cases/  1487 controls) | **OR per SD (95% CI) WHI** ^b,c^ |
| --- | --- | --- | --- | --- | --- | --- | --- | --- | --- | --- | --- | --- | --- | --- | --- |
| Age at menarche |  | |  |  | | |  | | | | | 0.96  (0.90–1.02) | 0.94  (0.86–1.02) | 1.00  (0.99-1.02) | 1.00  (1.00-1.00) |
| All–GECCO | 5832/6285 | | 1.08 | | 89.7% | | | 65.1% | | 33.1% | 11.1% |  |  |  |  |
| All–CORECT | 7112/4456 | | 0.63 | | 88.1% | | | 62.9% | | 31.7% | 10.8% |  |  |  |  |
| Combined | 12944/10741 | | 0.89 | | 99.5% | | | 90.6% | | 56.6% | 17.8% |  |  |  |  |
| Age at menopause | | 937/762 |  | | |  | | |  | | | 0.99  (0.97–1.01) | 0.95  (0.86–1.05) | 1.01  (0.99-1.03) | 1.00  (0.98-1.02) |
| All–GECCO | 5832/6285 | | 1.08 | | 83.2% | | | 56.7% | | 28.1% | 9.9% |  |  |  |  |
| All–CORECT | 7112/4456 | | 0.63 | | 79.5% | | | 52.7% | | 25.9% | 9.3% |  |  |  |  |
| Combined | 12944/10741 | | 0.89 | | 98.3% | | | 84.4% | | 48.7% | 15.3% |  |  |  |  |

Abbreviations: BMI, body mass index; CI, confidence interval; CRC, colorectal cancer; DACHS, Darmkrebs: Chancen der Verhütung durch Screening; OR, odds ratio; WHI, Women`s Health Initiative.

^a^assumed odds ratio per standard deviation of the exposure variable, assume 5 % alpha level, and an R² of 0.069 for age at menarche and 0.057 for age at menopause, which corresponds to the variance in age at menarche/age at menopause explained by the SNPs used for this analyses^[1](#_ENREF_1), [2](#_ENREF_2)^.

^b^ models adjusted by age, BMI, menopausal hormone therapy, family history of CRC, NSAID use, smoking.

^c^ odds ratio and 95% confidence intervals for the association of age at menarche (per SD–1.5 years in DACHS and 1.4 years in WHI) and age at menopause (per SD–4.8 years in DACHS and 6.5 years in WHI) and risk of CRC calculated in the DACHS / WHI studies respectively (part of GECCO consortium).

Calculations were performed using the online power calculator for Mendelian Randomization based on the publication by Burgess et al. 2014 ^[3](#_ENREF_3)^

Supplementary Table 5. Genetic variants associated with age at menarche ^[1](#_ENREF_1)^ and availability in GECCO / CORECT dataset

|  | **SNP** | **Chr** | | **Position** | **Effect/ other**  **allele** | **BETA^a^** | **SE^a^** | **SNP available** | **LD_SNP used** | **Effect/ other allele** | | **LD R²** | | **D'** | | | | | | | | | |  |  |  |
| --- | --- | --- | --- | --- | --- | --- | --- | --- | --- | --- | --- | --- | --- | --- | --- | --- | --- | --- | --- | --- | --- | --- | --- | --- | --- | --- |
| 1 | rs2308069 | | 1 | 7421139 | C/CTG | 0.0286 | 0.0046 | No | rs845193 | A/G | 1 | | | | | 1 | | | | | | | |  |  |  |
| 2 | rs6678140 | | 1 | 8436802 | C/T | 0.0269 | 0.0041 | Yes |  |  |  | | | | |  | | | | | | | |  |  |  |
| 3 | rs34646499 | | 1 | 14138813 | CG/C | 0.0354 | 0.0057 | No | rs2744687 | T/G | 0.98 | | | | | 1 | | | | | | | |  |  |  |
| 4 | rs12125335 | | 1 | 21385436 | C/T | 0.0496 | 0.0063 | Yes |  |  |  | | | | |  | | | | | | | |  |  |  |
| 5 | rs141847393 | | 1 | 27212209 | T/C | 0.0396 | 0.0071 | Yes |  |  |  | | | | |  | | | | | | | |  |  |  |
| 6 | rs360495 | | 1 | 33228197 | A/T | 0.0429 | 0.0072 | Yes |  |  |  | | | | |  | | | | | | | |  |  |  |
| 7 | rs4970598 | | 1 | 38940215 | T/C | 0.0624 | 0.0112 | Yes |  |  |  | | | | |  | | | | | | | |  |  |  |
| 8 | rs11209331 | | 1 | 41456689 | T/C | 0.0238 | 0.0039 | Yes |  |  |  | | | | |  | | | | | | | |  |  |  |
| 9 | rs11210871 | | 1 | 44029353 | C/G | 0.04 | 0.0042 | Yes |  |  |  | | | | |  | | | | | | | |  |  |  |
| 10 | rs643428 | | 1 | 54728858 | C/T | 0.0219 | 0.004 | Yes |  |  |  | | | | |  | | | | | | | |  |  |  |
| 11 | rs11357467 | | 1 | 65818709 | TA/T | 0.0255 | 0.0042 | No | rs7354899 | T/C | 1 | | | | | 1 | | | | | | | |  |  |  |
| 12 | rs11209943 | | 1 | 72750500 | A/G | 0.0384 | 0.0039 | Yes |  |  |  | | | | |  | | | | | | | |  |  |  |
| 13 | rs1040070 | | 1 | 74977870 | C/G | 0.05 | 0.0039 | Yes |  |  |  | | | |  | | | | | | | | |  |  |  |
| 14 | rs10782777 | | 1 | 82562929 | G/A | 0.025 | 0.0041 | Yes |  |  |  | | | | |  | | | | | | | |  |  |  |
| 15 | rs7517629 | | 1 | 91189933 | G/A | 0.0238 | 0.004 | No | rs12089815 | G/A | 0.90 | | | | | 0.99 | | | | | | | |  |  |  |
| 16 | rs11165924 | | 1 | 98375448 | A/G | 0.0312 | 0.0041 | Yes |  |  |  | | | | |  | | | | | | | |  |  |  |
| 17 | rs4561063 | | 1 | 102520898 | T/G | 0.0312 | 0.0039 | Yes |  |  |  | | | | |  | | | | | | | |  |  |  |
| 18 | rs61817552 | | 1 | 150423577 | G/A | 0.0285 | 0.0048 | Yes |  |  |  | | | | |  | | | | | | | |  |  |  |
| 19 | rs6661100 | | 1 | 150758727 | T/C | 0.0471 | 0.0067 | Yes |  |  |  | | | | |  | | | | | | | |  |  |  |
| 20 | rs4845364 | | 1 | 154141908 | A/G | 0.0215 | 0.0038 | Yes |  |  |  | | | | |  | | | | | | | |  |  |  |
| 21 | rs2661339 | | 1 | 163018934 | T/G | 0.0534 | 0.0088 | Yes |  |  |  | | | | |  | | | | | | | |  |  |  |
| 22 | rs157877 | | 1 | 165398744 | G/A | 0.0843 | 0.0058 | Yes |  |  |  | | | | |  | | | | | | | |  |  |  |
| 23 | rs61826838 | | 1 | 174028022 | C/T | 0.0322 | 0.0054 | No | rs714624 | A/G | 0.91 | | | | | 0.98 | | | | | | | |  |  |  |
| 24 | rs506589 | | 1 | 177894287 | T/C | 0.0695 | 0.0048 | Yes |  |  |  | | | | |  | | | | | | | |  |  |  |
| 25 | rs61828391 | | 1 | 179732142 | G/A | 0.0317 | 0.0058 | Yes |  |  |  | | | | |  | | | | | | | |  |  |  |
| 26 | rs58989029 | | 1 | 199798339 | G/GA | 0.0325 | 0.004 | No | rs6427782 | A/G | 1.00 | | | | | 1.00 | | | | | | | |  |  |  |
| 27 | rs11240695 | | 1 | 204158132 | C/A | 0.0333 | 0.0044 | Yes |  |  |  | | | | |  | | | | | | | |  |  |  |
| 28 | rs4951261 | | 1 | 205717823 | A/C | 0.0269 | 0.0039 | Yes |  |  |  | | | | |  | | | | | | | |  |  |  |
| 29 | rs12040029 | | 1 | 213451958 | C/T | 0.0392 | 0.0063 | Yes |  |  |  | | | | |  | | | | | | | |  |  |  |
| 30 | rs62104180 | | 2 | 466003 | A/G | 0.1134 | 0.0101 | Yes |  |  |  | | | | |  | | | | | | | |  |  |  |
| 31 | rs7576624 | | 2 | 625029 | C/T | 0.0741 | 0.005 | Yes |  |  |  | | | | |  | | | | | | | |  |  |  |
| 32 | rs7587651 | | 2 | 10368606 | C/T | 0.0238 | 0.004 | Yes |  |  |  | | | | |  | | | | | | | |  |  |  |
| 33 | rs150821390 | | 2 | 24106445 | T/C | 0.0717 | 0.0124 | Yes |  |  |  | | | | |  | | | | | | | |  |  |  |
| 34 | rs11461653 | | 2 | 25159172 | G/GC | 0.0252 | 0.0041 | No | rs6728219 | T/C | 1.00 | | | | | 1.00 | | | | | | | |  |  |  |
| 35 | rs72787511 | | 2 | 32816089 | C/G | 0.0646 | 0.0114 | Yes |  |  |  | | | | |  | | | | | | | |  |  |  |
| 36 | rs10175423 | | 2 | 42970161 | C/T | 0.0247 | 0.0042 | Yes |  |  |  | | | | |  | | | | | | | |  |  |  |
| 37 | rs17390720 | | 2 | 44952254 | C/G | 0.0259 | 0.0044 | Yes |  |  |  | | | |  | | | | | | | | |  |  |  |
| 38 | rs149544395 | | 2 | 48016353 | CTT/CT | 0.034 | 0.005 | No | rs3136269 | A/G | 0.98 | | | 0.99 | | | | | | | | | |  |  |  |
| 39 | rs111567162 | | 2 | 56587749 | A/T | 0.0675 | 0.0051 | No | rs6747380 | A/G | 0.92 | | | 0.99 | | | | | | | | | |  |  |  |
| 40 | rs1025128 | | 2 | 60175475 | G/C | 0.0219 | 0.0039 | Yes |  |  |  | | |  | | | | | | | | | |  |  |  |
| 41 | rs10205969 | | 2 | 61367664 | C/T | 0.0391 | 0.0054 | Yes |  |  |  | | |  | | | | | | | | | |  |  |  |
| 42 | rs12467441 | | 2 | 61685826 | C/T | 0.0408 | 0.006 | Yes |  |  |  | | |  | | | | | | | | | |  |  |  |
| 43 | rs2723065 | | 2 | 65279414 | G/A | 0.0247 | 0.0039 | Yes |  |  |  | | |  | | | | | | | | | |  |  |  |
| 44 | rs2312205 | | 2 | 69704941 | A/G | 0.0302 | 0.005 | Yes |  |  |  | | |  | | | | | | | | | |  |  |  |
| 45 | rs34437050 | | 2 | 73535526 | A/G | 0.2406 | 0.0204 | Yes |  |  |  | | |  | | | | | | | | | |  |  |  |
| 46 | rs2679894 | | 2 | 105870779 | A/G | 0.0505 | 0.0042 | Yes |  |  |  | | |  | | | | | | | | | |  |  |  |
| 47 | rs2558101 | | 2 | 137613322 | G/A | 0.0236 | 0.0043 | Yes |  |  |  | | |  | | | | | | | | | |  |  |  |
| 48 | rs35935052 | | 2 | 142302503 | T/G | 0.0437 | 0.0054 | Yes |  |  |  | | |  | | | | | | | | | |  |  |  |
| 49 | rs6434162 | | 2 | 153556801 | G/A | 0.0361 | 0.0052 | Yes |  |  |  | | |  | | | | | | | | | |  |  |  |
| 50 | rs142058842 | | 2 | 156621725 | G/C | 0.0681 | 0.0051 | Yes |  |  |  | | |  | | | | | | | | | |  |  |  |
| 51 | rs145438026 | | 2 | 157228255 | C/T | 0.0699 | 0.008 | Yes |  |  |  | | |  | | | | | | | | | |  |  |  |
| 52 | rs72186109 | | 2 | 164530192 | CACA/C | 0.0326 | 0.0045 | No | rs4667698 | T/C | 1 | | | 1 | | | | | | | | | |  |  |  |
| 53 | rs2271758 | | 2 | 172701157 | G/T | 0.0214 | 0.0039 | Yes |  |  |  | | |  | | | | | | | | | |  |  |  |
| 54 | rs842567 | | 2 | 184291116 | C/A | 0.034 | 0.005 | Yes |  |  |  | | |  | | | | | | | | | |  |  |  |
| 55 | rs10931831 | | 2 | 199621641 | C/T | 0.0531 | 0.004 | Yes |  |  |  | | |  | | | | | | | | | |  |  |  |
| 56 | rs13023912 | | 2 | 199756278 | G/A | 0.0508 | 0.004 | Yes |  |  |  | | |  | | | | | | | | | |  |  |  |
| 57 | rs16841867 | | 2 | 203168235 | C/G | 0.0456 | 0.006 | Yes |  |  |  | | |  | | | | | | | | | |  |  |  |
| 58 | rs184033703 | | 2 | 206956138 | G/A | 0.0479 | 0.0085 | Yes |  |  |  | | |  | | | | | | | | | |  |  |  |
| 59 | rs138546321 | | 2 | 209607565 | TTA/T | 0.0569 | 0.0065 | No | rs73068377 | T/A | 1 | | | 1 | | | | | | | | | |  |  |  |
| 60 | rs6735626 | | 2 | 213403972 | A/G | 0.0215 | 0.0039 | Yes |  |  |  | | |  | | | | | | | | | |  |  |  |
| 61 | rs73820560 | | 3 | 1906245 | C/A | 0.0319 | 0.0057 | Yes |  |  |  | | |  | | | | | | | | | |  |  |  |
| 62 | rs9867904 | | 3 | 18442437 | G/C | 0.0278 | 0.004 | Yes |  |  |  | | |  | | | | | | | | | |  |  |  |
| 63 | rs73035994 | | 3 | 24206463 | C/T | 0.0907 | 0.0116 | Yes |  |  |  | | |  | | | | | | | | | |  |  |  |
| 64 | rs1984870 | | 3 | 24715135 | T/G | 0.0422 | 0.0039 | Yes |  |  |  | | |  | | | | | | | | | |  |  |  |
| 65 | rs77955256 | | 3 | 44883523 | T/A | 0.036 | 0.0062 | Yes |  |  |  | | |  | | | | | | | | | |  |  |  |
| 66 | rs6803264 | | 3 | 49254427 | T/C | 0.0296 | 0.0046 | Yes |  |  |  | | |  | | | | | | | | | |  |  |  |
| 67 | rs115435316 | | 3 | 49568181 | A/G | 0.1146 | 0.0112 | Yes |  |  |  | | |  | | | | | | | | | |  |  |  |
| 68 | rs6445624 | | 3 | 51358019 | A/G | 0.0422 | 0.0056 | Yes |  |  |  | | |  | | | | | | | | | |  |  |  |
| 69 | rs10933 | | 3 | 52719816 | C/T | 0.0244 | 0.004 | Yes |  |  |  | | |  | | | | | | | | | |  |  |  |
| 70 | rs7431217 | | 3 | 68595634 | T/C | 0.0229 | 0.0039 | Yes |  |  |  | | |  | | | | | | | | | |  |  |  |
| 71 | rs7426534 | | 3 | 84462073 | G/A | 0.0237 | 0.0042 | Yes |  |  |  | | |  | | | | | | | | | |  |  |  |
| 72 | rs9758500 | | 3 | 86910329 | G/A | 0.0457 | 0.004 | Yes |  |  |  | | |  | | | | | | | | | |  |  |  |
| 73 | rs4859001 | | 3 | 88221517 | T/C | 0.0449 | 0.006 | Yes |  |  |  | | |  | | | | | | | | | |  |  |  |
| 74 | rs709488 | | 3 | 107700952 | C/A | 0.0217 | 0.0038 | Yes |  |  |  | | |  | | | | | | | | | |  |  |  |
| 75 | rs9834893 | | 3 | 114574749 | G/C | 0.0508 | 0.0074 | Yes |  |  |  | | |  | | | | | | | | | |  |  |  |
| 76 | rs10934420 | | 3 | 117552111 | C/T | 0.0546 | 0.0038 | Yes |  |  |  | | |  | | | | | | | | | |  |  |  |
| 77 | rs2461794 | | 3 | 127870060 | A/G | 0.0342 | 0.0043 | Yes |  |  |  | | |  | | | | | | | | | |  |  |  |
| 78 | rs6439371 | | 3 | 132610752 | G/A | 0.0304 | 0.004 | Yes |  |  |  | | |  | | | | | | | | | |  |  |  |
| 79 | rs6439713 | | 3 | 137128815 | A/C | 0.0259 | 0.0041 | Yes |  |  |  | | |  | | | | | | | | | |  |  |  |
| 80 | rs11711674 | | 3 | 156532953 | T/C | 0.0216 | 0.0038 | Yes |  |  |  | | |  | | | | | | | | | |  |  |  |
| 81 | rs13322435 | | 3 | 156795468 | A/G | 0.0357 | 0.004 | Yes |  |  |  | | |  | | | | | | | | | |  |  |  |
| 82 | rs582780 | | 3 | 172121443 | A/G | 0.0267 | 0.0039 | Yes |  |  |  | | |  | | | | | | | | | |  |  |  |
| 83 | rs7649124 | | 3 | 184030827 | C/G | 0.033 | 0.0045 | Yes |  |  |  | | |  | | | | | | | | | |  |  |  |
| 84 | rs2300922 | | 3 | 185651469 | T/C | 0.0432 | 0.0039 | Yes |  |  |  | | |  | | | | | | | | | |  |  |  |
| 85 | rs2108753 | | 4 | 3266860 | T/C | 0.0284 | 0.0038 | Yes |  |  |  | | |  | | | | | | | | | |  |  |  |
| 86 | rs4340786 | | 4 | 28746246 | A/T | 0.037 | 0.0043 | Yes |  |  |  | | |  | | | | | | | | | |  |  |  |
| 87 | rs4588499 | | 4 | 45910674 | G/A | 0.0235 | 0.0039 | Yes |  |  |  | | |  | | | | | | | | | |  |  |  |
| 88 | rs3113862 | | 4 | 95143122 | G/A | 0.0373 | 0.0039 | Yes |  |  |  | | |  | | | | | | | | | |  |  |  |
| 89 | rs3733632 | | 4 | 104640935 | G/A | 0.0536 | 0.0052 | Yes |  |  |  | | |  | | | | | | | | | |  |  |  |
| 90 | rs62342064 | | 4 | 104665972 | T/C | 0.0565 | 0.0065 | Yes |  |  |  | | |  | | | | | | | | | |  |  |  |
| 91 | rs115260227 | | 4 | 104774698 | G/A | 0.1621 | 0.0241 | Yes |  |  |  | | |  | | | | | | | | | |  |  |  |
| 92 | rs17035311 | | 4 | 106066293 | A/C | 0.036 | 0.0054 | Yes |  |  |  | | |  | | | | | | | | | |  |  |  |
| 93 | rs62316795 | | 4 | 132621869 | A/C | 0.0351 | 0.0053 | Yes |  |  |  | | |  | | | | | | | | | |  |  |  |
| 94 | rs13120031 | | 4 | 177465182 | T/C | 0.0274 | 0.0041 | Yes |  |  |  | | |  | | | | | | | | | |  |  |  |
| 95 | rs10521021 | | 5 | 35030311 | G/T | 0.024 | 0.0041 | Yes |  |  |  | | |  | | | | | | | | | |  |  |  |
| 96 | rs62361685 | | 5 | 41994067 | T/C | 0.0537 | 0.0085 | Yes |  |  |  | | |  | | | | | | | | | |  |  |  |
| 97 | rs7712046 | | 5 | 43134968 | C/T | 0.0333 | 0.0042 | Yes |  |  |  | | |  | | | | | | | | | |  |  |  |
| 98 | rs813301 | | 5 | 52909927 | T/C | 0.0274 | 0.0039 | Yes |  |  |  | | | | | | |  | | | | | |  |  |  |
| 99 | rs256350 | | 5 | 59140876 | C/T | 0.0238 | 0.0043 | Yes |  |  |  | | | | | | |  | | | | | |  |  |  |
| 100 | rs80170948 | | 5 | 64020316 | G/T | 0.0685 | 0.0109 | Yes |  |  |  | | | | | | |  | | | | | |  |  |  |
| 101 | rs13173441 | | 5 | 76982410 | T/C | 0.0331 | 0.0059 | No | rs1422409 | A/C | 0.99 | | | 1 | | | | | | | | | |  |  |  |
| 102 | rs17085593 | | 5 | 95630705 | C/G | 0.0246 | 0.0042 | Yes |  |  |  | | |  | | | | | | | | | |  |  |  |
| 103 | rs2916578 | | 5 | 107318454 | G/A | 0.0248 | 0.004 | No | rs288194 | T/C | 0.95 | | | 1 | | | | | | | | | |  |  |  |
| 104 | rs654354 | | 5 | 110503301 | T/A | 0.0233 | 0.0039 | Yes |  |  |  | | |  | | | | | | | | | |  |  |  |
| 105 | rs247520 | | 5 | 110876057 | T/C | 0.0361 | 0.0045 | Yes |  |  |  | | |  | | | | | | | | | |  |  |  |
| 106 | rs1566385 | | 5 | 111130474 | A/G | 0.0595 | 0.0084 | Yes |  |  |  | | |  | | | | | | | | | |  |  |  |
| 107 | rs62379978 | | 5 | 133915969 | G/T | 0.0638 | 0.0054 | Yes |  |  |  | | |  | | | | | | | | | |  |  |  |
| 108 | rs3815212 | | 5 | 137761555 | T/C | 0.0337 | 0.0046 | Yes |  |  |  | | |  | | | | | | | | | |  |  |  |
| 109 | rs6878910 | | 5 | 138281261 | A/G | 0.0381 | 0.0061 | Yes |  |  |  | | |  | | | | | | | | | |  |  |  |
| 110 | rs975642 | | 5 | 139384490 | C/T | 0.0245 | 0.0039 | Yes |  |  |  | | |  | | | | | | | | | |  |  |  |
| 111 | rs1428120 | | 5 | 153541904 | T/G | 0.025 | 0.0038 | Yes |  |  |  | | |  | | | | | | | | | |  |  |  |
| 112 | rs437836 | | 5 | 156715068 | T/C | 0.035 | 0.0052 | Yes |  |  |  | | |  | | | | | | | | | |  |  |  |
| 113 | rs34371367 | | 5 | 165941519 | AT/A | 0.0233 | 0.0042 | No | rs1823492 | G/A | 0.92 | | | 0.98 | | | | | | | | | |  |  |  |
| 114 | rs9647570 | | 5 | 167370263 | G/T | 0.0363 | 0.0056 | Yes |  |  |  | | |  | | | | | | | | | |  |  |  |
| 115 | rs2546959 | | 5 | 167404411 | T/G | 0.032 | 0.0053 | Yes |  |  |  | | |  | | | | | | | | | |  |  |  |
| 116 | rs4976623 | | 5 | 167947996 | C/G | 0.0296 | 0.0049 | Yes |  |  |  | | |  | | | | | | | | | |  |  |  |
| 117 | rs6864818 | | 5 | 168734867 | T/C | 0.0364 | 0.0046 | Yes |  |  |  | | |  | | | | | | | | | |  |  |  |
| 118 | rs4701140 | | 5 | 179034260 | A/G | 0.0242 | 0.004 | Yes |  |  |  | | |  | | | | | | | | | |  |  |  |
| 119 | rs2770957 | | 5 | 180656734 | C/G | 0.0323 | 0.0047 | Yes |  |  |  | | |  | | | | | | | | | |  |  |  |
| 120 | rs446745 | | 6 | 14918298 | C/T | 0.0258 | 0.0046 | Yes |  |  |  | | |  | | | | | | | | | |  |  |  |
| 121 | rs6927679 | | 6 | 18559687 | T/C | 0.027 | 0.0043 | Yes |  |  |  | | |  | | | | | | | | | |  |  |  |
| 122 | rs1539310 | | 6 | 22562485 | A/G | 0.0244 | 0.0045 | Yes |  |  |  | | |  | | | | | | | | | |  |  |  |
| 123 | rs12663002 | | 6 | 28441634 | T/C | 0.0427 | 0.0057 | Yes |  |  |  | | |  | | | | | | | | | |  |  |  |
| 124 | rs62391851 | | 6 | 29740548 | G/A | 0.0595 | 0.0085 | Yes |  |  |  | | |  | | | | | | | | | |  |  |  |
| 125 | rs3021057 | | 6 | 32652363 | T/C | 0.0249 | 0.0044 | Yes |  |  |  | | |  | | | | | | | | | |  |  |  |
| 126 | rs9349203 | | 6 | 41893323 | G/A | 0.0395 | 0.0038 | Yes |  |  |  | | |  | | | | | | | | | |  |  |  |
| 127 | rs79541760 | | 6 | 50930848 | A/T | 0.0368 | 0.0051 | Yes |  |  |  | | |  | | | | | | | | | |  |  |  |
| 128 | rs222440 | | 6 | 52946320 | C/T | 0.0325 | 0.005 | Yes |  |  |  | | |  | | | | | | | | | |  |  |  |
| 129 | rs9474996 | | 6 | 54642129 | T/A | 0.0343 | 0.0038 | No | rs12199827 | C/A | 1 | | | 1 | | | | | | | | | |  |  |  |
| 130 | rs9382676 | | 6 | 56859084 | T/C | 0.0373 | 0.0046 | Yes |  |  |  | | |  | | | | | | | | | |  |  |  |
| 131 | rs7753896 | | 6 | 76347020 | A/G | 0.031 | 0.0039 | Yes |  |  |  | | |  | | | | | | | | | |  |  |  |
| 132 | rs7757654 | | 6 | 77173780 | C/T | 0.0306 | 0.0042 | Yes |  |  |  | | |  | | | | | | | | | |  |  |  |
| 133 | rs1414186 | | 6 | 77713859 | G/T | 0.0432 | 0.0048 | Yes |  |  |  | | |  | | | | | | | | | |  |  |  |
| 134 | rs11756746 | | 6 | 84286477 | A/G | 0.0248 | 0.0045 | Yes |  |  |  | | |  | | | | | | | | | |  |  |  |
| 135 | rs6931884 | | 6 | 100158873 | T/C | 0.0592 | 0.0057 | Yes |  |  |  | | |  | | | | | | | | | |  |  |  |
| 136 | rs13199764 | | 6 | 100744134 | T/C | 0.039 | 0.0045 | Yes |  |  |  | | |  | | | | | | | | | |  |  |  |
| 137 | rs12200565 | | 6 | 100983589 | T/C | 0.0316 | 0.0039 | Yes |  |  |  | | |  | | | | | | | | | |  |  |  |
| 138 | rs395962 | | 6 | 105397418 | T/G | 0.1266 | 0.0041 | Yes |  |  |  | | |  | | | | | | | | | |  |  |  |
| 139 | rs6911407 | | 6 | 108867031 | A/C | 0.0291 | 0.0039 | Yes |  |  |  | | |  | | | | | | | | | |  |  |  |
| 140 | rs235696 | | 6 | 124253495 | C/G | 0.0225 | 0.004 | Yes |  |  |  | | |  | | | | | | | | | |  |  |  |
| 141 | rs4897178 | | 6 | 126712247 | T/G | 0.0426 | 0.0039 | No | rs1578060 | G/C | 0.99 | | | 1 | | | | | | | | | |  |  |  |
| 142 | rs4327718 | | 6 | 128364709 | G/A | 0.0329 | 0.0052 | Yes |  |  |  | | |  | | | | | | | | | |  |  |  |
| 143 | rs78928932 | | 6 | 136228617 | C/T | 0.0554 | 0.0091 | Yes |  |  |  | | |  | | | | | | | | | |  |  |  |
| 144 | rs117530880 | | 6 | 146687748 | G/T | 0.0658 | 0.0118 | Yes |  |  |  | | |  | | | | | | | | | |  |  |  |
| 145 | rs6911527 | | 6 | 148285329 | T/C | 0.0269 | 0.0045 | Yes |  |  |  | | |  | | | | | | | | | |  |  |  |
| 146 | rs6933660 | | 6 | 151803754 | C/A | 0.0343 | 0.0041 | Yes |  |  |  | | |  | | | | | | | | | |  |  |  |
| 147 | rs910425 | | 6 | 170652191 | G/A | 0.0223 | 0.004 | Yes |  |  |  | | |  | | | | | | | | | |  |  |  |
| 148 | rs10268051 | | 7 | 27763590 | A/C | 0.0249 | 0.0045 | Yes |  |  |  | | |  | | | | | | | | | |  |  |  |
| 149 | rs17171852 | | 7 | 41392815 | C/A | 0.038 | 0.0048 | Yes |  |  |  | | |  | | | | | | | | | |  |  |  |
| 150 | rs1079866 | | 7 | 41470093 | G/C | 0.0711 | 0.0056 | Yes |  |  |  | | |  | | | | | | | | | |  |  |  |
| 151 | rs1470750 | | 7 | 50576648 | G/C | 0.0223 | 0.0039 | Yes |  |  |  | | |  | | | | | | | | | |  |  |  |
| 152 | rs2267812 | | 7 | 74138121 | A/C | 0.0417 | 0.0049 | Yes |  |  |  | | |  | | | | | | | | | |  |  |  |
| 153 | rs187760798 | | 7 | 75099040 | T/C | 0.0576 | 0.0072 | No | rs55688682 | T/G | 0.99 | | | 1 | | | | | | | | | |  |  |  |
| 154 | rs1030015 | | 7 | 78139581 | G/T | 0.0206 | 0.0038 | Yes |  |  |  | | |  | | | | | | | | | |  |  |  |
| 155 | rs149226155 | | 7 | 93206613 | G/A | 0.0238 | 0.0041 | No | rs982692 | T/C | 0.98 | | | 0.99 | | | | | | | | | |  |  |  |
| 156 | rs15671 | | 7 | 94186064 | C/A | 0.0233 | 0.0038 | Yes |  |  |  | | |  | | | | | | | | | |  |  |  |
| 157 | rs999885 | | 7 | 99701176 | A/G | 0.0241 | 0.0038 | Yes |  |  |  | | |  | | | | | | | | | |  |  |  |
| 158 | rs1456031 | | 7 | 114296102 | T/C | 0.0216 | 0.0039 | Yes |  |  |  | | |  | | | | | | | | | |  |  |  |
| 159 | rs10237306 | | 7 | 121955981 | T/G | 0.0296 | 0.004 | Yes |  |  |  | | |  | | | | | | | | | |  |  |  |
| 160 | rs11767400 | | 7 | 122160742 | A/C | 0.0289 | 0.0042 | Yes |  |  |  | | |  | | | | | | | | | |  |  |  |
| 161 | rs11556924 | | 7 | 129663496 | T/C | 0.0227 | 0.0041 | Yes |  |  |  | | | |  | | | |  |  |  |  |  |  |  |  |
| 162 | rs17563472 | | 7 | 130409054 | C/T | 0.0606 | 0.0107 | Yes |  |  |  | | |  | | | | | | | | | | | | |
| 163 | rs12707076 | | 7 | 132729814 | C/G | 0.0273 | 0.004 | Yes |  |  |  | | |  | | | | | | | | | | | | |
| 164 | rs13233916 | | 7 | 138874416 | G/C | 0.0496 | 0.0075 | Yes |  |  |  | | |  | | | | | | | | | | | | |
| 165 | rs7004265 | | 8 | 1526356 | T/C | 0.023 | 0.0039 | No | rs7015811 | T/C | 0.94 | | | 0.98 | | | | | | | | | | | | |
| 166 | rs2688326 | | 8 | 3767623 | C/T | 0.0364 | 0.0042 | Yes |  |  |  | | |  | | | | | | | | | | | | |
| 167 | rs2724961 | | 8 | 4560227 | C/T | 0.0459 | 0.0038 | Yes |  |  |  | | |  | | | | | | | | | | | | |
| 168 | rs4875424 | | 8 | 4831685 | C/T | 0.0332 | 0.004 | Yes |  |  |  | | |  | | | | | | | | | | | | |
| 169 | rs6185 | | 8 | 25280800 | G/C | 0.0301 | 0.0044 | Yes |  |  |  | | |  | | | | | | | | | | | | |
| 170 | rs13278754 | | 8 | 34902952 | G/C | 0.0251 | 0.0042 | Yes |  |  |  | | |  | | | | | | | | | | | | |
| 171 | rs4487799 | | 8 | 53163528 | A/T | 0.0241 | 0.0042 | Yes |  |  |  | | |  | | | | | | | | | | | | |
| 172 | rs16918378 | | 8 | 53877882 | T/C | 0.0478 | 0.0059 | Yes |  |  |  | | |  | | | | | | | | | | | | |
| 173 | rs1449543 | | 8 | 76591987 | T/C | 0.0224 | 0.0038 | Yes |  |  |  | | |  | | | | | | | | | | | | |
| 174 | rs10094506 | | 8 | 78097161 | C/T | 0.0454 | 0.0043 | No | rs4735761 | A/C | 1 | | | 1 | | | | | | | |  |  |  |  |  |
| 175 | rs35485457 | | 8 | 78679087 | G/T | 0.0359 | 0.0042 | Yes |  |  |  | | |  | | | | | | | | | |  |  |  |
| 176 | rs7465046 | | 8 | 87245253 | C/T | 0.0409 | 0.0046 | No | rs4310184 | T/C | 0.89 | | | 0.97 | | | | | | | | | |  |  |  |
| 177 | rs2441873 | | 8 | 105329096 | T/G | 0.0235 | 0.0039 | No | rs2514653 | T/C | 1 | | | 1 | | | | | | | | | |  |  |  |
| 178 | rs7826872 | | 8 | 132071766 | T/C | 0.0262 | 0.004 | Yes |  |  |  | | |  | | | | | | | | | |  |  |  |
| 179 | rs2542420 | | 8 | 140645701 | C/G | 0.0327 | 0.004 | Yes |  |  |  | | |  | | | | | | | | | |  |  |  |
| 180 | rs552491 | | 9 | 1711210 | G/A | 0.0294 | 0.004 | Yes |  |  |  | | |  | | | | | | | | | |  |  |  |
| 181 | rs913588 | | 9 | 7174673 | G/A | 0.0339 | 0.0038 | Yes |  |  |  | | |  | | | | | | | | | |  |  |  |
| 182 | rs10959016 | | 9 | 10283451 | G/A | 0.0306 | 0.0048 | Yes |  |  |  | | |  | | | | | | | | | |  |  |  |
| 183 | rs10959552 | | 9 | 11130009 | G/A | 0.0371 | 0.0059 | Yes |  |  |  | | |  | | | | | | | | | |  |  |  |
| 184 | rs1601615 | | 9 | 11813745 | C/T | 0.028 | 0.0039 | Yes |  |  |  | | |  | | | | | | | | | |  |  |  |
| 185 | rs7849973 | | 9 | 22819576 | C/G | 0.024 | 0.004 | Yes |  |  |  | | |  | | | | | | | | | |  |  |  |
| 186 | rs1329767 | | 9 | 73798371 | C/A | 0.0292 | 0.004 | Yes |  |  |  | | |  | | | | | | | | | |  |  |  |
| 187 | rs2604265 | | 9 | 76905178 | A/G | 0.0386 | 0.0043 | Yes |  |  |  | | |  | | | | | | | | | |  |  |  |
| 188 | rs35436838 | | 9 | 77273910 | G/T | 0.0678 | 0.0105 | Yes |  |  |  | | |  | | | | | | | | | |  |  |  |
| 189 | rs2378100 | | 9 | 80513323 | C/T | 0.0237 | 0.0038 | Yes |  |  |  | | |  | | | | | | | | | |  |  |  |
| 190 | rs4877387 | | 9 | 81679875 | T/C | 0.0241 | 0.0043 | Yes |  |  |  | | |  | | | | | | | | | |  |  |  |
| 191 | rs11534296 | | 9 | 83282402 | G/A | 0.0368 | 0.0043 | Yes |  |  |  | | |  | | | | | | | | | |  |  |  |
| 192 | rs7853970 | | 9 | 86715566 | T/C | 0.0449 | 0.0039 | Yes |  |  |  | | |  | | | | | | | | | |  |  |  |
| 193 | rs1571536 | | 9 | 92215638 | T/C | 0.0331 | 0.0038 | Yes |  |  |  | | |  | | | | | | | | | |  |  |  |
| 194 | rs9330454 | | 9 | 92515514 | G/A | 0.0307 | 0.0042 | Yes |  |  |  | | |  | | | | | | | | | |  |  |  |
| 195 | rs10992769 | | 9 | 96276910 | C/G | 0.0286 | 0.0042 | Yes |  |  |  | | |  | | | | | | | | | |  |  |  |
| 196 | rs10156597 | | 9 | 108941509 | A/T | 0.1024 | 0.0041 | Yes |  |  |  | | |  | | | | | | | | | |  |  |  |
| 197 | rs11792861 | | 9 | 111809295 | A/C | 0.032 | 0.0042 | Yes |  |  |  | | |  | | | | | | | | | |  |  |  |
| 198 | rs7852169 | | 9 | 114318394 | G/C | 0.0973 | 0.0068 | Yes |  |  |  | | |  | | | | | | | | | |  |  |  |
| 199 | rs2780243 | | 9 | 120730928 | C/T | 0.0234 | 0.0039 | Yes |  |  |  | | |  | | | | | | | | | |  |  |  |
| 200 | rs4836984 | | 9 | 127405632 | T/C | 0.0342 | 0.0038 | Yes |  |  |  | | |  | | | | | | | | | |  |  |  |
| 201 | rs467379 | | 9 | 136905474 | T/C | 0.0239 | 0.0042 | Yes |  |  |  | | |  | | | | | | | | | |  |  |  |
| 202 | rs10978641 | | 9 | 109554196 | T/A | 0.0318 | 0.0046 | Yes |  |  |  | | |  | | | | | | | | | |  |  |  |
| 203 | rs7907759 | | 10 | 1730008 | A/G | 0.0409 | 0.0039 | Yes |  |  |  | | |  | | | | | | | | | |  |  |  |
| 204 | rs7912468 | | 10 | 2697434 | C/T | 0.0239 | 0.0039 | Yes |  |  |  | | |  | | | | | | | | | |  |  |  |
| 205 | rs1885740 | | 10 | 10251910 | G/A | 0.0256 | 0.0046 | Yes |  |  |  | | |  | | | | | | | | | |  |  |  |
| 206 | rs10906395 | | 10 | 13541008 | C/T | 0.0233 | 0.0039 | Yes |  |  |  | | |  | | | | | | | | | |  |  |  |
| 207 | rs61846901 | | 10 | 51056858 | C/T | 0.0257 | 0.0042 | Yes |  |  |  | | |  | | | | | | | | | |  |  |  |
| 208 | rs6415872 | | 10 | 63660689 | A/G | 0.0236 | 0.0039 | No |  |  |  | | |  | | | | | | | | | |  |  |  |
| 209 | rs5785580 | | 10 | 65191645 | A/AT | 0.0281 | 0.004 | Yes | rs7924036 | T/G | 0.98 | | | 0.99 | | | | | | | | | |  |  |  |
| 210 | rs7072571 | | 10 | 71380093 | A/G | 0.0306 | 0.0055 | Yes |  |  |  | | |  | | | | | | | | | |  |  |  |
| 211 | rs4746113 | | 10 | 74071178 | G/A | 0.0244 | 0.0042 | Yes |  |  |  | | |  | | | | | | | | | |  |  |  |
| 212 | rs77532868 | | 10 | 88081438 | T/C | 0.0573 | 0.0097 | No |  |  |  | | |  | | | | | | | | | |  |  |  |
| 213 | rs10617761 | | 10 | 90255578 | C/CAG | 0.031 | 0.0055 | Yes | rs12572235 | T/C | 1 | | | 1 | | | | | | | | | |  |  |  |
| 214 | rs1172955 | | 10 | 97877320 | T/A | 0.044 | 0.0043 | Yes |  |  |  | | |  | | | | | | | | | |  |  |  |
| 215 | rs72842141 | | 10 | 102686073 | T/A | 0.0618 | 0.009 | Yes |  |  |  | | |  | | | | | | | | | |  |  |  |
| 216 | rs59543819 | | 10 | 103754188 | C/T | 0.025 | 0.0042 | Yes |  |  |  | | |  | | | | | | | | | |  |  |  |
| 217 | rs2066323 | | 10 | 104871361 | G/A | 0.0237 | 0.0039 | Yes |  |  |  | | |  | | | | | | | | | |  |  |  |
| 218 | rs10885077 | | 10 | 112759731 | T/G | 0.0241 | 0.0044 | No |  |  |  | | |  | | | | | | | | | |  |  |  |
| 219 | rs11349289 | | 10 | 117484684 | CA/C | 0.028 | 0.0042 | Yes | rs11197420 | G/A | 1 | | | 1 | | | | | | | | | |  |  |  |
| 220 | rs4751614 | | 10 | 118696266 | A/T | 0.0297 | 0.0046 | Yes |  |  |  | | |  | | | | | | | | | |  |  |  |
| 221 | rs10400136 | | 10 | 120833948 | G/A | 0.0259 | 0.0039 | Yes |  |  |  | | |  | | | | | | | | | |  |  |  |
| 222 | rs73435048 | | 10 | 121154531 | G/A | 0.0441 | 0.0081 | Yes |  |  |  | | |  | | | | | | | | | |  |  |  |
| 223 | rs12571664 | | 10 | 121708929 | T/C | 0.0367 | 0.0048 | Yes |  |  |  | | | |  | | | | | | | |  |  |  |  |
| 224 | rs7077302 | | 10 | 123676662 | C/G | 0.0497 | 0.007 | Yes |  |  |  | | |  | | | | | | | | | |  |  |  |
| 225 | rs9422857 | | 10 | 126861278 | G/C | 0.0278 | 0.0038 | No |  |  |  | | |  | | | | | | | | | |  |  |  |
| 226 | rs4576738 | | 10 | 134295644 | A/G | 0.0266 | 0.0043 | Yes | rs7904728 | A/G | 0.92 | | | 0.98 | | | | | | | | | |  |  |  |
| 227 | rs3782120 | | 11 | 206089 | A/G | 0.0334 | 0.0044 | Yes |  |  |  | | |  | | | | | | | | | |  |  |  |
| 228 | rs16937956 | | 11 | 8404501 | G/A | 0.0383 | 0.004 | Yes |  |  |  | | |  | | | | | | | | | |  |  |  |
| 229 | rs10832021 | | 11 | 13324530 | G/A | 0.047 | 0.0042 | Yes |  |  |  | | |  | | | | | | | | | |  |  |  |
| 230 | rs4359170 | | 11 | 16596152 | A/T | 0.0282 | 0.0041 | Yes |  |  |  | | |  | | | | | | | | | |  |  |  |
| 231 | rs1032682 | | 11 | 22791324 | T/C | 0.0214 | 0.0038 | Yes |  |  |  | | |  | | | | | | | | | |  |  |  |
| 232 | rs16917237 | | 11 | 27702383 | T/G | 0.0393 | 0.0047 | No |  |  |  | | |  | | | | | | | | | |  |  |  |
| 233 | rs6484408 | | 11 | 28888653 | G/A | 0.0248 | 0.0043 | Yes | rs7948265 | A/C | 0.98 | | | 1 | | | | | | | | | |  |  |  |
| 234 | rs11031040 | | 11 | 30317733 | G/T | 0.0404 | 0.0052 | Yes |  |  |  | | |  | | | | | | | | | |  |  |  |
| 235 | rs1023955 | | 11 | 43608835 | G/T | 0.0279 | 0.0039 | Yes |  |  |  | | |  | | | | | | | | | |  |  |  |
| 236 | rs970179 | | 11 | 45433845 | A/G | 0.0213 | 0.0039 | Yes |  |  |  | | |  | | | | | | | | | |  |  |  |
| 237 | rs953230 | | 11 | 46064974 | A/G | 0.033 | 0.0042 | Yes |  |  |  | | |  | | | | | | | | | |  |  |  |
| 238 | rs68002803 | | 11 | 46539110 | T/C | 0.0267 | 0.0042 | No |  |  |  | | |  | | | | | | | | | |  |  |  |
| 239 | rs59265730 | | 11 | 47622412 | A/AAAAAAC | 0.0254 | 0.0041 | Yes | rs11039308 | A/G | 1 | | | 1 | | | | | | | | | |  |  |  |
| 240 | rs10897450 | | 11 | 63593219 | C/G | 0.0229 | 0.0038 | Yes |  |  |  | | |  | | | | | | | | | |  |  |  |
| 241 | rs10750766 | | 11 | 65473798 | C/A | 0.0278 | 0.0043 | Yes |  |  |  | | |  | | |  |  |  |  |  |  |  |  |  |  |
| 242 | rs7115444 | | 11 | 77555824 | T/C | 0.0333 | 0.0048 | Yes |  |  |  | | |  | | | | | | | | | | | |  |
| 243 | rs4945266 | | 11 | 78027488 | G/A | 0.0445 | 0.0052 | Yes |  |  |  | | |  | | | | | | | | | | | |  |
| 244 | rs4402316 | | 11 | 84752914 | C/G | 0.0313 | 0.0047 | Yes | rs11823251 | G/C | 0.93 | | | 0.98 | | | | | | | | | | | |  |
| 245 | rs7108556 | | 11 | 86716236 | T/C | 0.0312 | 0.0047 | Yes |  |  |  | | |  | | | | | | | | | | | |  |
| 246 | rs6590889 | | 11 | 101438191 | C/T | 0.0437 | 0.004 | Yes |  |  |  | | |  | | | | | | | | | | | |  |
| 247 | rs17564430 | | 11 | 115043574 | G/T | 0.035 | 0.0044 | Yes |  |  |  | | |  | | | | | | | | | | | |  |
| 248 | rs1815811 | | 11 | 119059404 | G/A | 0.027 | 0.0038 | Yes |  |  |  | | |  | | | | | | | | | | | |  |
| 249 | rs7114175 | | 11 | 122813983 | T/A | 0.0599 | 0.0038 | Yes |  |  |  | | |  | | | | | | | | | | | |  |
| 250 | rs77530428 | | 12 | 17126283 | G/A | 0.1241 | 0.0165 | Yes |  |  |  | | |  | | | | | | | | | | | |  |
| 251 | rs10842343 | | 12 | 24579079 | T/A | 0.0256 | 0.0039 | Yes |  |  |  | | |  | | | | | | | | | | | |  |
| 252 | rs7971408 | | 12 | 47876942 | T/C | 0.0453 | 0.0063 | Yes |  |  |  | | |  | | | | | | | | | | | |  |
| 253 | rs1054442 | | 12 | 49389320 | A/C | 0.0355 | 0.004 | Yes |  |  |  | | |  | | | | | | | | | | | |  |
| 254 | rs7132908 | | 12 | 50263148 | G/A | 0.0424 | 0.0039 | Yes |  |  |  | | |  | | | | | | | | | | | |  |
| 255 | rs1131017 | | 12 | 56435929 | C/G | 0.0229 | 0.0039 | Yes |  |  |  | | |  | | | | | | | | | | | |  |
| 256 | rs1148006 | | 12 | 75978358 | G/A | 0.0259 | 0.0044 | No |  |  |  | | |  | | | | | | | | | | | |  |
| 257 | rs9943694 | | 12 | 84199793 | A/G | 0.0285 | 0.005 | Yes | rs55686872 | T/A | 0.97 | | | 1 | | | | | | | | | | | |  |
| 258 | rs7979001 | | 12 | 97506357 | A/G | 0.0219 | 0.0038 | Yes |  |  |  | | |  | | | | | | | | | | | |  |
| 259 | rs3764002 | | 12 | 108618630 | C/T | 0.0304 | 0.0045 | Yes |  |  |  | | |  | | | | | | | | | | | |  |
| 260 | rs11065822 | | 12 | 111600134 | T/G | 0.0258 | 0.0043 | Yes |  |  |  | | |  | | | | | | | | | | | |  |
| 261 | rs474463 | | 12 | 115107376 | C/T | 0.0291 | 0.0048 | No |  |  |  | | |  | | | | | | | | | | | |  |
| 262 | rs7133066 | | 12 | 117353716 | T/G | 0.0334 | 0.0057 | Yes | rs7484620 | A/G | 0.94 | | | 0.99 | | | | | | | | | | | |  |
| 263 | rs660549 | | 12 | 121300988 | C/T | 0.0212 | 0.0038 | No |  |  |  | | |  | | | | | | | | | | | |  |
| 264 | rs5801963 | | 12 | 132412133 | AG/A | 0.026 | 0.0047 | Yes | rs3923717 | A/G | 0.98 | | | 1 | | | | | | | | | | | |  |
| 265 | rs9548873 | | 13 | 40238492 | C/T | 0.0311 | 0.004 | Yes |  |  |  | | |  | | | | | | | | | | | |  |
| 266 | rs73187215 | | 13 | 42646769 | G/A | 0.0377 | 0.0066 | Yes |  |  |  | | |  | | | | | | | | | | | |  |
| 267 | rs9568123 | | 13 | 49475780 | G/A | 0.0291 | 0.0053 | Yes |  |  |  | | |  | | | | | | | | | | | |  |
| 268 | rs4886140 | | 13 | 59833519 | A/G | 0.0284 | 0.0041 | Yes |  |  |  | | |  | | | | | | | | | | | |  |
| 269 | rs1925047 | | 13 | 74600274 | C/A | 0.0341 | 0.0041 | Yes |  |  |  | | |  | | | | | | | | | | | |  |
| 270 | rs11619721 | | 13 | 112082513 | G/T | 0.0413 | 0.0073 | Yes |  |  |  | | |  | | | | | | | | | | | |  |
| 271 | rs9522262 | | 13 | 112186283 | C/G | 0.0411 | 0.0039 | Yes |  |  |  | | |  | | | | | | | | | | | |  |
| 272 | rs74499585 | | 13 | 112285043 | A/G | 0.0583 | 0.0079 | Yes |  |  |  | | |  | | | | | | | | | | | |  |
| 273 | rs10136330 | | 14 | 30514335 | C/T | 0.0579 | 0.01 | Yes |  |  |  | | |  | | | | | | | | | | | |  |
| 274 | rs35035068 | | 14 | 40965279 | AT/A | 0.0257 | 0.0041 | No | rs17110826 | T/C | 1 | | | 1 | | | | | | | | | | | |  |
| 275 | rs10138913 | | 14 | 60943106 | T/C | 0.056 | 0.0041 | Yes |  |  |  | | |  | | | | | | | | | | | |  |
| 276 | rs10143972 | | 14 | 93850179 | C/T | 0.0388 | 0.0049 | Yes |  |  |  | | |  | | | | | | | | | | | |  |
| 277 | rs10145469 | | 14 | 97769834 | C/A | 0.0609 | 0.0093 | Yes |  |  |  | | |  | | | | | | | | | | | |  |
| 278 | rs941520 | | 14 | 99709702 | C/A | 0.0223 | 0.0039 | Yes |  |  |  | | |  | | | | | | | | | | | |  |
| 279 | rs12894936 | | 14 | 100846991 | C/T | 0.0522 | 0.0043 | Yes |  |  |  | | |  | | | | | | | | | | | |  |
| 280 | rs6575806 | | 14 | 101353211 | C/A | 0.0334 | 0.0058 | Yes |  |  |  | | |  | | | | | | | | | | | |  |
| 281 | rs79084266 | | 14 | 101367407 | G/C | 0.0404 | 0.0074 | Yes |  |  |  | | |  | | | | | | | | | | | |  |
| 282 | rs7178532 | | 15 | 23794517 | A/G | 0.0439 | 0.0041 | Yes |  |  |  | | | |  | | | | | |  |  |  |  |  |  |
| 283 | rs184950120 | | 15 | 23810843 | C/T | 0.3961 | 0.0555 | Yes |  |  |  | | |  | | | | | | | | | | | |  |
| 284 | rs8040272 | | 15 | 24824016 | A/G | 0.0441 | 0.0062 | Yes |  |  |  | | |  | | | | | | | | | | | |  |
| 285 | rs34513772 | | 15 | 40608820 | T/C | 0.0244 | 0.0042 | Yes |  |  |  | | |  | | | | | | | | | | | |  |
| 286 | rs4924538 | | 15 | 41469313 | TTT/!TTT | 0.0255 | 0.0042 | No | rs537244 | T/G | 0.83 | | | 0.97 | | | | | | | | | | | |  |
| 287 | rs1435753 | | 15 | 47925066 | C/T | 0.0278 | 0.004 | Yes |  |  |  | | |  | | | | | | | | | | | |  |
| 288 | rs28757192 | | 15 | 51507610 | C/T | 0.0627 | 0.0106 | Yes |  |  |  | | | |  | | | | | |  |  |  |  |  |  |
| 289 | rs11852771 | | 15 | 54364552 | A/G | 0.0242 | 0.004 | Yes |  |  |  | | |  | | | | | | | | | |  |  |  |
| 290 | rs3743266 | | 15 | 60781513 | T/C | 0.0416 | 0.004 | Yes |  |  |  | | |  | | | | | | | | | |  |  |  |
| 291 | rs72756954 | | 15 | 64537300 | C/G | 0.0577 | 0.0081 | Yes |  |  |  | | |  | | | | | | | | | |  |  |  |
| 292 | rs10153031 | | 15 | 67937755 | T/G | 0.0403 | 0.0039 | No | rs11637027 | G/T | 0.99 | | | 1 | | | | | | | | | |  |  |  |
| 293 | rs5742915 | | 15 | 74336633 | C/T | 0.0234 | 0.0038 | Yes |  |  |  | | |  | | | | | | | | | |  |  |  |
| 294 | rs35510813 | | 15 | 77802791 | TA/T | 0.0264 | 0.0045 | No | rs11857450 | G/C | 1 | | | 1 | | | | | | | | | |  |  |  |
| 295 | rs1971554 | | 15 | 83406228 | T/C | 0.032 | 0.0044 | Yes |  |  |  | | |  | | | | | | | | | |  |  |  |
| 296 | rs12915845 | | 15 | 89042467 | C/T | 0.0403 | 0.0039 | Yes |  |  |  | | |  | | | | | | | | | |  |  |  |
| 297 | rs148825694 | | 15 | 99302408 | T/TATC | 0.0564 | 0.0097 | No | rs72769809 | T/C | 0.98 | | | 1 | | | | | | | | | |  |  |  |
| 298 | rs758747 | | 16 | 3627358 | C/T | 0.0268 | 0.0043 | Yes |  |  |  | | |  | | | | | | | | | |  |  |  |
| 299 | rs1704528 | | 16 | 14388750 | C/T | 0.0508 | 0.0041 | Yes |  |  |  | | |  | | | | | | | | | |  |  |  |
| 300 | rs153793 | | 16 | 15542199 | G/A | 0.0234 | 0.0038 | Yes |  |  |  | | |  | | | | | | | | | |  |  |  |
| 301 | rs112991346 | | 16 | 19967668 | C/T | 0.0446 | 0.0055 | Yes |  |  |  | | |  | | | | | | | | | |  |  |  |
| 302 | rs4780885 | | 16 | 20380004 | C/G | 0.0258 | 0.0038 | Yes |  |  |  | | |  | | | | | | | | | |  |  |  |
| 303 | rs113388806 | | 16 | 24804954 | T/A | 0.0624 | 0.0103 | Yes |  |  |  | | |  | | | | | | | | | |  |  |  |
| 304 | rs8051833 | | 16 | 29896390 | G/A | 0.0404 | 0.0042 | Yes |  |  |  | | |  | | | | | | | | | |  |  |  |
| 305 | rs3809624 | | 16 | 30102802 | C/T | 0.0302 | 0.0043 | Yes |  |  |  | | |  | | | | | | | | | |  |  |  |
| 306 | rs143461173 | | 16 | 52279707 | A/G | 0.0288 | 0.005 | No | rs12599115 | A/T | 1 | | | 1 | | | | | | | | | |  |  |  |
| 307 | rs9972653 | | 16 | 53814363 | G/T | 0.0509 | 0.0039 | Yes |  |  |  | | |  | | | | | | | | | |  |  |  |
| 308 | rs7359336 | | 16 | 69733460 | G/A | 0.0534 | 0.0038 | Yes |  |  |  | | |  | | | | | | | | | |  |  |  |
| 309 | rs4448948 | | 16 | 72569236 | T/A | 0.0411 | 0.0075 | Yes |  |  |  | | |  | | | | | | | | | |  |  |  |
| 310 | rs112752732 | | 17 | 1942577 | C/G | 0.0572 | 0.0099 | Yes |  |  |  | | |  | | | | | | | | | |  |  |  |
| 311 | rs142643995 | | 17 | 2017993 | T/C | 0.0646 | 0.0117 | Yes |  |  |  | | |  | | | | | | | | | |  |  |  |
| 312 | rs12603280 | | 17 | 6034754 | G/A | 0.037 | 0.0045 | Yes |  |  |  | | |  | | | | | | | | | |  |  |  |
| 313 | rs55680968 | | 17 | 7774047 | G/A | 0.0455 | 0.0075 | Yes |  |  |  | | |  | | | | | | | | | |  |  |  |
| 314 | rs59246405 | | 17 | 43123625 | T/C | 0.0274 | 0.0039 | Yes |  |  |  | | |  | | | | | | | | | |  |  |  |
| 315 | rs11079810 | | 17 | 46227846 | T/C | 0.0358 | 0.0062 | Yes |  |  |  | | |  | | | | | | | | | |  |  |  |
| 316 | rs9635759 | | 17 | 49613785 | A/G | 0.059 | 0.0041 | Yes |  |  |  | | |  | | | | | | | | | |  |  |  |
| 317 | rs2787487 | | 17 | 53209382 | C/G | 0.0311 | 0.0039 | Yes |  |  |  | | |  | | | | | | | | | |  |  |  |
| 318 | rs7218751 | | 17 | 77796437 | A/G | 0.0333 | 0.0049 | Yes |  |  |  | | |  | | | | | | | | | |  |  |  |
| 319 | rs59652033 | | 17 | 77951023 | C/T | 0.0263 | 0.0043 | Yes |  |  |  | | |  | | | | | | | | | |  |  |  |
| 320 | rs66508321 | | 17 | 78739672 | G/A | 0.0303 | 0.0041 | Yes |  |  |  | | |  | | | | | | | | | |  |  |  |
| 321 | rs2659007 | | 17 | 79217478 | G/A | 0.0306 | 0.0039 | Yes |  |  |  | | |  | | | | | | | | | |  |  |  |
| 322 | rs12937034 | | 17 | 79446015 | G/A | 0.0254 | 0.0041 | Yes |  |  |  | | |  | | | | | | | | | |  |  |  |
| 323 | rs11873906 | | 18 | 3813464 | G/A | 0.0507 | 0.0043 | Yes |  |  |  | | |  | | | | | | | | | |  |  |  |
| 324 | rs8087304 | | 18 | 31765736 | A/T | 0.0222 | 0.0038 | Yes |  |  |  | |  | | | | | | | | | | | |  |  |
| 325 | rs1512238 | | 18 | 44748467 | G/A | 0.0537 | 0.0038 | Yes |  |  |  | | |  | | | | | | | | | |  |  |  |
| 326 | rs7239114 | | 18 | 45921214 | G/A | 0.022 | 0.004 | Yes |  |  |  | | |  | | | | | | | | | |  |  |  |
| 327 | rs3746037 | | 19 | 1828948 | A/C | 0.0361 | 0.0048 | Yes |  |  |  | | |  | | | | | | | | | |  |  |  |
| 328 | rs169080 | | 19 | 4980864 | C/T | 0.0259 | 0.004 | Yes |  |  |  | | |  | | | | | | | | | |  |  |  |
| 329 | rs484353 | | 19 | 7891767 | A/G | 0.0316 | 0.0039 | Yes |  |  |  | | |  | | | | | | | | | |  |  |  |
| 330 | rs4804117 | | 19 | 9950127 | T/G | 0.0455 | 0.0039 | No | rs8104651 | T/C | 0.91 | | | 0.98 | | | | | | | | | |  |  |  |
| 331 | rs10422323 | | 19 | 13104027 | A/G | 0.037 | 0.0062 | Yes |  |  |  | | | |  | | | | | | | | |  |  |  |
| 332 | rs12460047 | | 19 | 18346228 | G/A | 0.0283 | 0.0043 | Yes |  |  |  | | | | | | | | |  | | | | | | |
| 333 | rs11668587 | | 19 | 18829770 | G/A | 0.0329 | 0.0041 | Yes |  |  |  | | | | | | | | |  | | | | | | |
| 334 | rs56367141 | | 19 | 31051857 | C/A | 0.0401 | 0.006 | Yes |  |  |  | | | | | | | | |  | | | | | | |
| 335 | rs29941 | | 19 | 34309532 | A/G | 0.0278 | 0.0041 | Yes |  |  |  | | | | | | | | |  | | | | | | |
| 336 | rs77563037 | | 19 | 36204690 | C/CG | 0.033 | 0.004 | No | rs107068 | G/A | 1 | | | 1 | | | | |  |  |  |  |  |  |  |  |
| 337 | rs4804025 | | 19 | 47609223 | G/A | 0.0409 | 0.0042 | Yes |  |  |  | | |  | | | | | | | | | |  |  |  |
| 338 | rs2548458 | | 19 | 49207651 | T/C | 0.0212 | 0.0038 | Yes | rs570794 | C/T | 0.98 | | | 1 | | | | | | | | | |  |  |  |
| 339 | rs4801809 | | 19 | 50334895 | C/T | 0.0435 | 0.0072 | Yes |  |  |  | | |  | | | | | | | | | |  |  |  |
| 340 | rs2889128 | | 19 | 58973929 | A/C | 0.0222 | 0.0038 | Yes |  |  |  | | |  | | | | | | | | | |  |  |  |
| 341 | rs852061 | | 20 | 17109159 | C/A | 0.0365 | 0.0039 | Yes |  |  |  | | |  | | | | | | | | | |  |  |  |
| 342 | rs1535252 | | 20 | 19682834 | C/T | 0.0252 | 0.0038 | Yes |  |  |  | | |  | | | | | | | | | |  |  |  |
| 343 | rs111558392 | | 20 | 20346178 | C/T | 0.0404 | 0.0055 | No | rs6046818 | T/C | 0.99 | | | 1 | | | | | | | | | |  |  |  |
| 344 | rs4813429 | | 20 | 21485806 | T/C | 0.0307 | 0.0052 | Yes |  |  |  | | |  | | | | | | | | | |  |  |  |
| 345 | rs1737894 | | 20 | 31054702 | C/G | 0.022 | 0.0039 | Yes |  |  |  | | |  | | | | | | | | | |  |  |  |
| 346 | rs2295094 | | 20 | 33447915 | A/G | 0.0364 | 0.0053 | Yes |  |  |  | | |  | | | | | | | | | |  |  |  |
| 347 | rs36093651 | | 20 | 37287102 | T/C | 0.0371 | 0.0046 | Yes |  |  |  | | |  | | | | | | | | | |  |  |  |
| 348 | rs2425674 | | 20 | 43529461 | G/C | 0.0215 | 0.0038 | Yes |  |  |  | | |  | | | | | | | | | |  |  |  |
| 349 | rs3746619 | | 20 | 54823805 | A/C | 0.0475 | 0.0069 | Yes |  |  |  | | |  | | | | | | | | | |  |  |  |
| 350 | rs13043968 | | 20 | 54830983 | C/A | 0.0413 | 0.0062 | Yes |  |  |  | | |  | | | | | | | | | |  |  |  |
| 351 | rs443252 | | 20 | 62799680 | T/C | 0.0591 | 0.0092 | Yes |  |  |  | | |  | | | | | | | | | |  |  |  |
| 352 | rs62229372 | | 21 | 37692507 | T/C | 0.051 | 0.0061 | Yes |  |  |  | | |  | | | | | | | | | |  |  |  |
| 353 | rs117143374 | | 21 | 40555561 | C/T | 0.0502 | 0.0057 | Yes |  |  |  | | |  | | | | | | | | | |  |  |  |
| 354 | rs151680 | | 22 | 22273242 | T/C | 0.028 | 0.0038 | Yes |  |  |  | | |  | | | | | | | | | |  |  |  |
| 355 | rs5753377 | | 22 | 31293700 | G/A | 0.0306 | 0.0043 | Yes |  |  |  | | |  | | | | | | | | | |  |  |  |
| 356 | rs4303811 | | 22 | 39157755 | G/A | 0.0353 | 0.006 | Yes |  |  |  | | |  | | | | | | | | | |  |  |  |
| 357 | rs9614460 | | 22 | 45745229 | G/T | 0.0249 | 0.0041 | Yes |  |  |  | | |  | | | | | | | | | |  |  |  |
| 358 | rs8136272 | | 22 | 49678782 | A/T | 0.04 | 0.0044 | Yes |  |  |  | | |  | | | | | | | | | |  |  |  |

Abbreviations: Chr, chromosome, LD, linkage disequilibrium; SE, standard error; SNP, single nucleotide polymorphism.

Supplementary Table 6. List of 51 SNPs associated with age at menopause ^[2](#_ENREF_2)^

|  | **SNP** | **Chr** | **Position** | **Effect/ other allele** | **Effect** | **SE** |
| --- | --- | --- | --- | --- | --- | --- |
| 1 | rs4246511 | 1 | 39152972 | T/C | 0.2181 | 0.0232 |
| 2 | rs12142240 | 1 | 46519888 | C/T | 0.1267 | 0.0218 |
| 3 | rs1411478 | 1 | 179228905 | G/A | 0.1316 | 0.0205 |
| 4 | rs2236918 | 1 | 240084449 | G/C | 0.1529 | 0.0205 |
| 5 | rs704795 | 2 | 27569998 | G/A | 0.1634 | 0.0206 |
| 6 | rs1800932 | 2 | 47871585 | G/A | 0.1731 | 0.0261 |
| 7 | rs930036 | 2 | 171649264 | G/A | 0.1852 | 0.0207 |
| 8 | rs16858210 | 3 | 185106704 | A/G | 0.1412 | 0.0238 |
| 9 | rs4693089 | 4 | 84592646 | G/A | 0.2022 | 0.0206 |
| 10 | rs6856693 | 4 | 185985800 | G/A | 0.1626 | 0.021 |
| 11 | rs427394 | 5 | 6798875 | A/G | 0.1264 | 0.0215 |
| 12 | rs11738223 | 5 | 171867097 | G/A | 0.1234 | 0.022 |
| 13 | rs2241584 | 5 | 175888783 | G/A | 0.1394 | 0.0207 |
| 14 | rs365132 | 5 | 176311180 | T/G | 0.2424 | 0.0201 |
| 15 | rs6899676 | 6 | 11003246 | G/A | 0.2289 | 0.0254 |
| 16 | rs9393800 | 6 | 11059723 | A/G | 0.1689 | 0.0232 |
| 17 | rs707938 | 6 | 31837338 | A/G | 0.1691 | 0.0217 |
| 18 | rs12196873 | 6 | 111704751 | C/A | 0.1618 | 0.0291 |
| 19 | rs2720044 | 8 | 38099744 | C/A | 0.29 | 0.0302 |
| 20 | rs10957156 | 8 | 61791955 | G/A | 0.1387 | 0.0237 |
| 21 | rs4879656 | 9 | 33002382 | C/A | 0.1188 | 0.0212 |
| 22 | rs10905065 | 10 | 5809833 | G/A | 0.1128 | 0.0205 |
| 23 | rs11031006 | 11 | 30183104 | A/G | 0.2165 | 0.029 |
| 24 | rs6484478 | 11 | 30263016 | A/G | 0.0992 | 0.0241 |
| 25 | rs10734411 | 11 | 32498360 | G/A | 0.1221 | 0.0205 |
| 26 | rs2277339 | 12 | 55432336 | T/G | 0.312 | 0.0346 |
| 27 | rs3741604 | 12 | 64982677 | C/T | 0.0918 | 0.0215 |
| 28 | rs7397861 | 12 | 65100733 | C/G | 0.0954 | 0.0212 |
| 29 | rs551087 | 12 | 119693576 | A/G | 0.1254 | 0.0228 |
| 30 | rs1727326 | 12 | 122166039 | G/C | 0.1948 | 0.0323 |
| 31 | rs12824058 | 12 | 129370287 | A/G | 0.1355 | 0.0207 |
| 32 | rs4886238 | 13 | 60011740 | A/G | 0.1768 | 0.0216 |
| 33 | rs1713460 | 14 | 20003455 | A/G | 0.144 | 0.0227 |
| 34 | rs9796 | 15 | 39058739 | A/T | 0.1309 | 0.0204 |
| 35 | rs1054875 | 15 | 87680130 | A/T | 0.1881 | 0.0208 |
| 36 | rs9039 | 16 | 9112864 | T/C | 0.1248 | 0.0226 |
| 37 | rs10852344 | 16 | 11924420 | C/T | 0.1646 | 0.0206 |
| 38 | rs12599106 | 16 | 34355526 | T/A | 0.1159 | 0.0209 |
| 39 | rs8070740 | 17 | 5272620 | G/A | 0.1465 | 0.0243 |
| 40 | rs2941505 | 17 | 35086230 | G/A | 0.1303 | 0.0217 |
| 41 | rs1799949 | 17 | 38498992 | A/G | 0.1393 | 0.0214 |
| 42 | rs349306 | 19 | 901694 | A/G | 0.2273 | 0.0356 |
| 43 | rs7259376 | 19 | 22299545 | G/A | 0.1106 | 0.0202 |
| 44 | rs11668344 | 19 | 60525476 | A/G | 0.4115 | 0.0211 |
| 45 | rs12461110 | 19 | 61012475 | G/A | 0.1744 | 0.0216 |
| 46 | rs451417 | 20 | 5889999 | C/A | 0.1954 | 0.0333 |
| 47 | rs16991615 | 20 | 5896227 | A/G | 0.8752 | 0.0436 |
| 48 | rs2236553 | 20 | 60760188 | T/C | 0.1574 | 0.0254 |
| 49 | rs13040088 | 20 | 61019647 | A/G | 0.1575 | 0.0249 |
| 50 | rs5762534 | 22 | 26963571 | C/T | 0.1636 | 0.0281 |
| 51 | rs763121 | 22 | 37209886 | A/G | 0.1645 | 0.0224 |

Abbreviations: Chr, chromosome, LD, linkage disequilibrium; SE, standard error; SNP, single nucleotide polymorphism.

Supplementary Table 7. Association between age at menarche/age at menopause and CRC risk using Mendelian randomization analyses and sensitivity analyses by GECCO and CORECT consortium and meta-analyzed results

|  |  | | |  | | **GRS-based analyses** | | | | **2-sample MR** | | | |
| --- | --- | --- | --- | --- | --- | --- | --- | --- | --- | --- | --- | --- | --- |
|  |  | | | | **Colorectal cancer^a^** | | **Adjusted by GRS-BMI^a,c^** | **Restricted risk score^a,d^** |  | | **MR-Egger^b,^** | **Weighted Median Estimator^b^** |  |
| Variable  (per year) | | N (cases/controls) | | | OR (95%CI) | | OR (95% CI) | OR (95% CI) |  | | OR (95% CI) | OR (95% CI) |  |
| ***Age at menarche*** | | | | |  | |  |  |  | |  |  |  |
| GECCO | | | 5832/6285 | | 0.97 (0.92–1.02) | | 0.98 (0.93–1.03) | 0.99 (0.94–1.04) |  | | 0.93 (0.74-1.18) | 0.94 (0.81–1.09) |  |
| CORECT | | | 7112/4456 | | 1.00 (0.95–1.05) | | 1.00 (0.95–1.05) | 1.00 (0.94–1.06) |  | | 1.05 (0.82-1.35) | 1.07 (0.92–1.24) |  |
| Meta-analysis | | | | | 0.98 (0.95–1.02) | | 0.99 (0.95–1.02) | 0.99 (0.95–1.03) |  | | 0.99 (0.83-1.17) | 1.00 (0.90–1.11) |  |
| ***Age at menopause*** | | | | |  | |  |  |  | |  |  |  |
| GECCO | | | 5832/6285 | | 0.99 (0.95-1.03) | | NA | NA |  | | 1.01 (0.91-1.12) | 0.99 (0.92–1.06) |  |
| CORECT | | | 7112/4456 | | 0.96 (0.92–1.01) | | NA | NA |  | | 1.03 (0.92-1.15) | 1.01 (0.94–1.09) |  |
| Meta-analysis | | | | | 0.98 (0.94–1.01) | |  |  |  | | 1.02 (0.94-1.10) | 1.00 (0.95–1.05) |  |
| ***Time period of hormone exposure*** | | | | |  | |  |  |  | |  |  |  |
| GECCO | | | 5832/6285 | | 1.01 (0.99–1.02) | | 1.00 (0.97–1.04) | 1.00 (0.97–1.02) |  | | 0.98 (0.93-1.03) | 0.99 (0.93–1.06) |  |
| CORECT | | | 7112/4456 | | 0.98 (0.96–1.00) | | 0.98 (0.94–1.01) | 0.98 (0.96–1.01) |  | | 0.96 (0.90-1.01) | 1.01 (0.94–1.08) |  |
| Meta-analysis | | | | | 0.99 (0.97–1.02) | | 0.99 (0.97–1.02) | 0.99 (0.97–1.01) |  | | 0.97 (0.93-1.01) | 1.00 (0.95–1.05) |  |

Abbreviations: BMI, body mass index; CCFR, colon cancer family registry; CI, confidence interval; GRS, genetic risk score; MR, Mendelian randomization; NA, not applicable; OR, odds ratio; SNP, single nucleotide polymorphism.

CCFR centers participated in GECCO or CORECT and were analyzed as such;
^a^ adjusted for age, study and principal components of genetic ancestry;
^b^ estimate derived using summary statistics; se for calculation of CI obtained via bootstrapping;
^c^ additionally adjusted for a genetic risk score for body mass index (GRS-BMI) out of 77 reported SNPs for BMI^[4](#_ENREF_4)^;
^d^ 42 BMI-associated SNPs were excluded from the age at menarche and time period of estrogen-exposure–risk scores.

**Supplementary Table 10.** Association between age at menarche/age at menopause and CRC risk
additionally adjusted by potential confounders in GECCO/CORECT

|  |  | | | **GRS-based analyses** | | | |
| --- | --- | --- | --- | --- | --- | --- | --- |
|  |  | | | | **MR-estimate^a^** |  | **Full model^a,b^** |
| Variable  (per year) | | N (cases/controls) | | | OR (95%CI) | N (cases/controls) | OR (95% CI) |
| ***Age at menarche*** | | | | |  |  |  |
| GECCO | | | 5832/6285 | | 0.97 (0.92–1.02) | 2820/2848 | 0.99 (0.92–1.07) |
| CORECT | | | 7112/4456 | | 1.00 (0.95–1.05) | 986/867 | 1.02 (0.89–1.16) |
| Meta-analysis | | | | | 0.98 (0.95–1.02) |  | 1.00 (0.93–1.06) |
| ***Age at menopause*** | | | | |  |  |  |
| GECCO | | | 5832/6285 | | 0.99 (0.95-1.03) | 2820/2848 | 0.96 (0.90-1.03) |
| CORECT | | | 7112/4456 | | 0.96 (0.92–1.01) | 986/867 | 0.96 (0.86-1.08) |
| Meta-analysis | | | | | 0.98 (0.94–1.01) |  | 0.96 (0.91-1.02) |
| ***Time period of hormone exposure*** | | | | |  |  |  |
| GECCO | | | 5832/6285 | | 1.01 (0.99–1.02) | 2820/2848 | 0.98 (0.96–1.01) |
| CORECT | | | 7112/4456 | | 0.98 (0.96–1.00) | 986/867 | 0.97 (0.93–1.01) |
| Meta-analysis | | | | | 0.99 (0.97–1.02) |  | 0.98 (0.94–1.02) |

Abbreviations: BMI, body mass index; CCFR, colon cancer family registry; CI, confidence interval; GRS, genetic risk score;

MHT, menopausal hormone therapy; MR, Mendelian randomization; OR, odds ratio; NA, not applicable;

SNP, single nucleotide polymorphism.

^a^ logistic regression model adjusted for age, study, principal components of genetic ancestry,

^b^ additionally adjusted for education, family history of CRC, ever regular aspirin use, MHT usage, BMI and smoking.


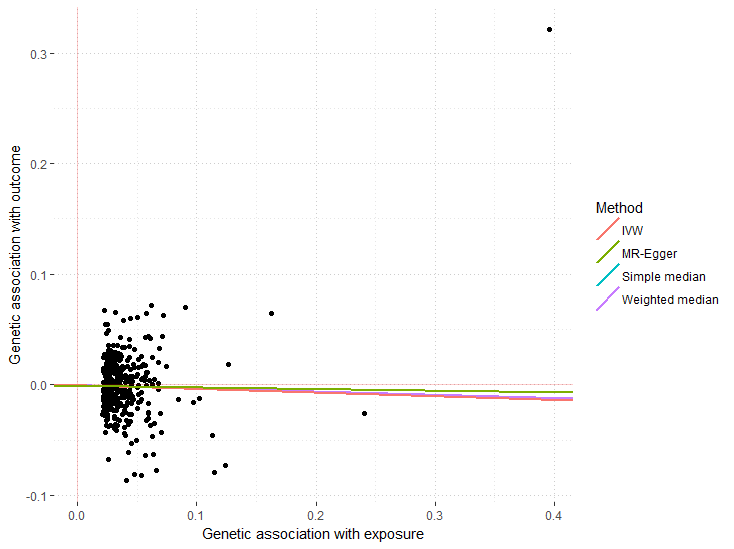


Supplementary Figure 1: Scatterplot of single-nucleotide polymorphism (SNP)-exposure associations on SNP-outcome associations for age at menarche on colorectal cancer (CRC) risk. The red line shows standard Mendelian randomization (MR) analysis inverse-variance weighted (IVW) estimator, where the ratio estimates (gene-exposure/gene-outcome associations) from each SNP are combined in an IVW estimate ^[5](#_ENREF_5)^, the green line shows MR-Egger regression ^[6](#_ENREF_6)^ and blue and violet lines show (simple and weighted) median regression ^[7](#_ENREF_7)^. The MR-Egger intercept of -0.0008 (95% confidence interval: -0.007, 0.005) yielded no indication of strong pleiotropic effects. This plot was drawn using the R-package “MendelianRandomization” [^8^](#_ENREF_8).


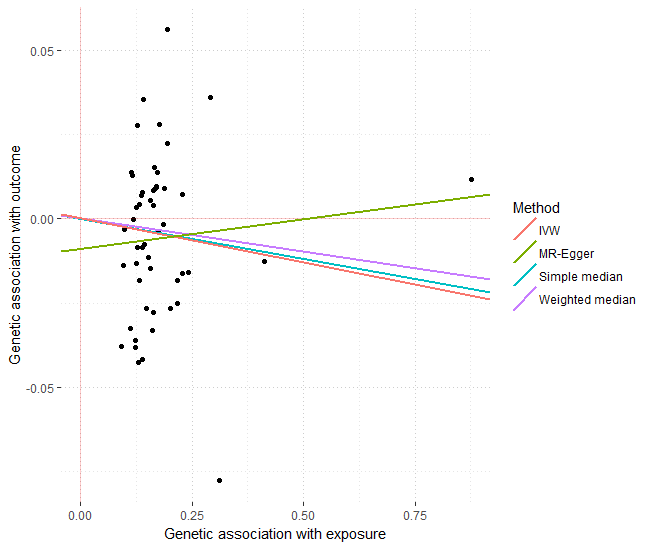


Supplementary Figure 2: Scatterplot of single-nucleotide polymorphism (SNP)-exposure associations on SNP-outcome associations for age at menopause on colorectal cancer (CRC) risk. The red line shows standard Mendelian randomization (MR) analysis inverse-variance weighted (IVW) estimator, where the ratio estimates (gene-exposure/gene-outcome associations) from each SNP are combined in an IVW estimate ^[5](#_ENREF_5)^ , the green line shows MR-Egger regression ^[6](#_ENREF_6)^ and blue and violet lines show (simple and weighted) median regression ^[7](#_ENREF_7)^. MR-Egger intercept of -0.009 (95% confidence interval: -0.023, 0.006) lies near zero and does not indicate large pleiotropic effects. This plot was drawn using the R-package “MendelianRandomization” [^8^](#_ENREF_8).


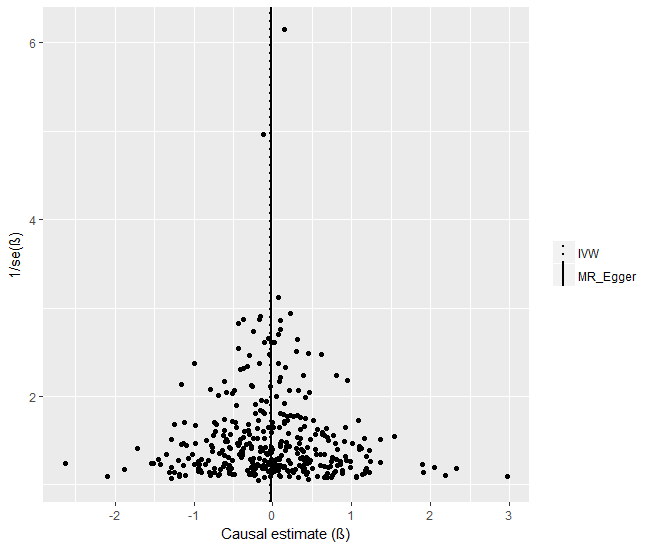


**Supplementary Figure 3: Funnelplot for causal estimates of age at menarche and colorectal cancer (CRC) risk.** The single SNP`s Mendelian randomization (MR) estimates (β) are plotted against 1/standard error (β). The overall causal estimates (beta coefficients) of the association of age at menarche on CRC risk are also shown for inverse-variance weighted estimator (IVW) estimator (dashed black line) and MR-Egger (solid black line).


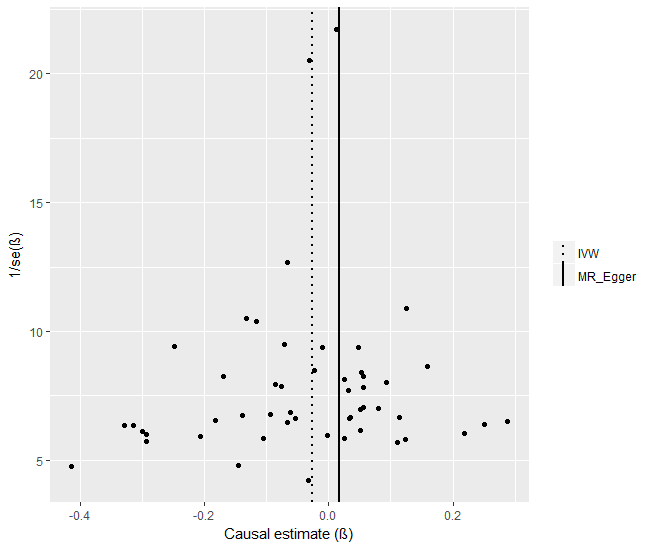


**Supplementary Figure 4: Funnelplot for causal estimates of age at menopause and colorectal cancer (CRC) risk**. The single SNP`s Mendelian randomization (MR) estimates (β) are plotted against 1/standard error (β). The overall causal estimates (beta coefficients) of the association of age at menarche on CRC risk are also shown for inverse-variance weighted estimator (IVW) estimator (dashed black line) and MR-Egger (solid black line).

**Supplementary Information on Genotyping/Imputation**

For GECCO genotype data was generated from germline DNA on Illumina 300K, Illumina OmniExpress, Illumina 550K/610K, or Affymetrix 100K/500K and Illumina Omni 2.5. For CCFR genotyping was done using Illumina 1M, 1M-Duo, or Omni1. CORECT samples were genotyped using germline DNA in Affymetrix Axiom and Illumina Infinium OncoArray-500K BeadChip (OncoArray); Illumina 1M, 1M-Duo, or Omni1; and Illlumina Omni 2.5 (Molecular Epidemiology of Colorectal Cancer Study (MECC)). A subset of additional CORECT samples has been genotyped on the OncoArray platform (described in the associated study descriptions). Imputation for all SNPs was conducted using the imputation panel of the Haplotype Reference Consortium.

**Supplementary Information on participant overlap**

There is some overlap between participants in GECCO/CORECT and the studies used for age at menarche/age at menopause GWAS. For age at menarche the studies NHS (12 081 participants in the REPROGEN consortium; 1343 controls and 806 cases in GECCO and 78 controls and 85 cases in CORECT), MCCS (412 participants in the REPROGEN consortium; 308 controls and 340 cases in CORECT) and SEARCH (10 184 participants in the REPROGEN consortium; 46 controls and 1778 cases in CORECT) were included in both REPROGEN consortium and GECCO/CORECT. These are 6.9% of the participants (n=329,345) of the REPROGEN consortium and 20.2% of the 23 685 participants (12 944 CRC cases and 10 741 controls) from GECCO/CORECT.

For age at menopause the NHS study was included both in the REPROGEN consortium (7024 participants, 10.1% of the 69360 participants included in the age at menopause GWAS) and in GECCO/CORECT (1343 controls and 806 cases in GECCO and 78 controls and 85 cases in CORECT; in total 9.8% of the 23 685 participants included in GECCO/CORECT).

**STUDIES**

**GECCO**

**The French Association Study Evaluating RISK for sporadic colorectal cancer (ASTERISK)**^[9](#_ENREF_9)^: Participants were recruited from the Pays de la Loire region in France between December 2002 and March 2006. Eligibility criteria for cases included being of Caucasian origin, being greater than or 40 years of age at diagnosis, and having no family history of colorectal cancer or polyps. Cases were patients with first primary colorectal cancer diagnosed in one of the six public hospitals and five clinics located in the Pays de la Loire region which participated in the study. Cases were confirmed based on medical and pathology reports. Controls were recruited at two Health Examination Centers of the Pays de la Loire region, and the recruitment of controls greater than or 70 years was completed in the departments of internal medicine and hepatogastroenterology of the University Hospital Center of Nantes, located in the same region. Controls were eligible to participate if they were Caucasian, aged greater than or 40 years, and had no family history of colorectal cancer or polyps. In the presence of the physician, each participant filled out a standardized questionnaire on family information, medical history, lifestyle, and dietary intake. Cases and controls provided a blood sample.

**Hawaii Colorectal Cancer Studies 2 and 3 (Colo2&3)** [^10^](#_ENREF_10)**:** Patients with colorectal cancer were identified through the rapid reporting system of the Hawaii SEER registry and consisted of all Japanese, Caucasian, and Native Hawaiian residents of Oahu who were newly diagnosed with an adenocarcinoma of the colon or rectum between January 1994 and August 1998. Control subjects were selected from participants in an on-going population-based health survey conducted by the Hawaii State Department of Health and from Health Care Financing Administration participants. Controls were matched to cases by sex, ethnicity, and age (within two years). Personal interviews were obtained from 768 matched pairs, resulting in a participation rate of 58.2% for cases and 53.2% for controls. A questionnaire, administered during an in-person interview, included questions about demographics, lifetime history of tobacco, alcohol use, aspirin use, physical activity, personal medical history, family history of colorectal cancer, height and weight, diet (FFQ), and postmenopausal hormone use. A blood sample was obtained from 548 (71%) of interviewed cases and 662 (86%) of interviewed controls. SEER staging information was extracted from the Hawaii Tumor Registry. In GECCO, self-reported Caucasian subjects with DNA, and clinical and epidemiologic data were selected for genotyping.

**Darmkrebs: Chancen der Verhütung durch Screening (DACHS)** [^11^](#_ENREF_11)^,^ [^12^](#_ENREF_12)**:** This German study was initiated as a large population-based case-control study in 2003 in the Rhine-Neckar-Odenwald region (southwest region of Germany) to assess the potential of endoscopic screening for reduction of colorectal cancer risk and to investigate etiologic determinants of disease, particularly lifestyle/environmental factors and genetic factors. Cases with a ﬁrst diagnosis of invasive colorectal cancer (ICO-10 codes C18-C20) who were at least 30 years of age (no upper age limit), German speaking, a resident in the study region, and mentally and physically able to participate in a one-hour interview, were recruited by their treating physicians either in the hospital a few days after surgery, or by mail after discharge from the hospital. Cases were conﬁrmed based on histologic reports and hospital discharge letters following diagnosis of colorectal cancer. All hospitals treating colorectal cancer patients in the study region participated. Based on estimates from population-based cancer registries, more than 50% of all potentially eligible patients with incident colorectal cancer in the study region were included. Community-based controls were randomly selected from population registries, employing frequency matching with respect to age (5-year groups), sex, and county of residence. Controls with a history of colorectal cancer were excluded. Controls were contacted by mail and follow-up calls. The participation rate was 51%. During an in-person interview, data were collected on demographics, medical history, family history of CRC, and various life-style factors, as were blood and mouthwash samples. The Set 1 scan consisted of a subset of participants recruited up to 2007, and samples were frequency matched on age and sex. The Set 2 scan consisted of additional subjects that were recruited up to 2010 as part of this ongoing study.

**Diet, Activity and Lifestyle Study (DALS)** [^13^](#_ENREF_13)**:** DALS is a population-based case-control study of colon cancer. Participants were recruited between 1991 and 1994 from three locations: the Kaiser Per-manente Medical Care Program (KPMCP) of Northern California, an eight-county area in Utah, and the metropolitan Twin Cities area of Minnesota. Eligibility criteria for cases included age at diagnosis between 30 and 79 years, diagnosis with ﬁrst primary colon cancer (ICD-O-2 codes 18.0 and 18.2-18.9) between October 1st 1991 and September 30th 1994, English speaking, and competency to complete the interview. Individuals with cancer of the rectosigmoid junction or rectum were excluded, as were those with a pathology report noting familial adenomatous polyposis, Crohns disease, or ulcerative colitis. A rapid-reporting system was used to identify all incident cases of colon cancer resulting in the majority of cases being interviewed within four months of diagnosis. Controls from KPMCP were randomly selected from membership lists. In Utah, controls under 65 years of age were randomly selected through random-digit dialing and driver license lists. Controls, 65 years of age and older, were randomly selected from Health Care Financing Administration lists. In Minnesota, controls were identiﬁed from Minnesota driver’s license or state ID lists. Cases and controls were matched by 5-year age groups and sex. The Set I scan consisted of a subset of the study designed above, from Utah, Minnesota, and KPMCP, and was restricted to subjects who self-reported as White non-Hispanic. The Set 2 scan consisted of subjects from Utah and Minnesota that were not genotyped in Set 1. Set 2 was restricted to subjects who self-reported as White non-Hispanic and those that had appropriate consent to post data to dbGaP.

**Multi-Ethnic Cohort Study (MEC)**[^14^](#_ENREF_14)**:** MEC was initiated in 1993 to investigate the impact of dietary and environmental factors on major chronic diseases, particularly cancer, in ethnically diverse populations in Hawai’i and California. The study recruited 96,810 men and 118,441 women aged 45 to 75 years between 1993 and 1996. Incident colorectal cancer cases occurring since January 1995, and controls were contacted for blood or saliva samples. The median interval between diagnosis and blood draw was 14 months (interquartile range, 10-19) among cases and the participation rate 74%. A sample of cohort participants was randomly selected to serve as controls at the onset of the nested case-control study (participation rate 66%). The selection was stratified by sex, age, and race/ethnicity. Colorectal cancer cases are identified through the Rapid Reporting System of the Hawai’i Tumor Registry and through quarterly linkage to the Los Angeles County Cancer Surveillance Program. Both registries are members of SEER. In GECCO, self-reported White subjects from the nested case-control study described above with DNA, and clinical and epidemiologic data were selected for genotyping.

**Nurses’ Health Study (NHS)** [^15^](#_ENREF_15)**:** The NHS cohort began in 1976 when 121,700 married female registered nurses aged 30 to 55 years returned the initial questionnaire that ascertained a variety of important health-related exposures. Since 1976, follow-up questionnaires have been mailed every two years. Colorectal cancer and other outcomes were reported by participants or next-of-kin and followed up through review of the medical and pathology record by physicians. Overall, more than 97% of self-reported colorectal cancers were conﬁrmed by medical-record review. Information was abstracted on histology and primary location. Follow-up has been high: as a proportion of the total possible follow-up time, follow-up has been over 92%. Colorectal cancer cases were ascertained through June 1, 2008. In 1989-90, 32,826 women in NHS I, mailed in blood samples by overnight courier which were aliquoted into buﬀy coat and stored in liquid nitrogen. In 2001-04, 29,684 women in NHS I who did not previously provide a blood sample mailed in a “swish-and-spit” sample of buccal cells. Incident cases are deﬁned as those occurring after the subject provided a blood or buccal sample. Prevalent cases are deﬁned as those occurring after enrollment in the study in 1976, but prior to the subject providing either a blood or buccal sample. After excluding participants with histories of cancer (except non-melanoma skin), ulcerative colitis, or familial polyposis, we constructed two case-control sets from which DNA was isolated from either buﬀy coat or buccal cells for genotyping: 1) a case-control set with cases of colorectal cancer matched to randomly selected controls who provided a blood sample and were free of colorectal cancer at the same time that the colorectal cancer was diagnosed in the case; 2) a case-control set with cases of colorectal cancer matched to randomly selected controls who provided a buccal sample and were free of colorectal cancer at the same time that the colorectal cancer was diagnosed in the cases. For both case-control sets, matching criteria included year of birth (within one year) and month/year of blood or buccal cell sampling (within six months). Cases were pair matched 1:1, 1:2, or 1:3 with a control participant(s).

**Ontario Familial Colorectal Cancer Registry** (**OFCCR)**: In GECCO, a subset of the Assessment of Risk in Colorectal Tumours in Canada (ARCTIC) from the Ontario Registry for Studies of Familial Colorectal Cancer (OFCCR) was used. Both the case-control study ^[16](#_ENREF_16)^ and the OFCCR ^[17](#_ENREF_17)^ have been described in detail previously, as have GWAS results ^[18](#_ENREF_18)^. In brief, cases were confirmed incident colorectal cancer (CRC) cases ages 20 to 74 years, residents of Ontario identified through comprehensive registry and diagnosed between July 1997 and June 2000. Population-based controls were randomly selected among Ontario residents (random-digit-dialing and listing of all Ontario residents), and matched by sex and 5-year age groups. A total of 1,236 CRC cases and 1,223 controls were successfully genotyped on at least one of the Illumina 1536 GoldenGate assay (Illumina, Inc, San Diego, CA), the Affymetrix GeneChip® Human Mapping 100K and 500K Array Set (Affymetrix, Inc, Santa Clara, CA), and a 10K non-synonymous SNP chip. Analysis was based on a set of unrelated subjects who were non-Hispanic, White by self-report or by investigation of genetic ancestry. We further excluded subjects if there was a sample mix-up, if they were missing epidemiologic questionnaire data, if they were cases with a tumor in the appendix, or if they were overlapped with the Colon Cancer Family Registry. Additionally, only samples genotyped on the Affymetrix GeneChip® 500K Array were utilized in order to avoid coverage issues in imputation.

**Prostate, Lung, Colorectal and Ovarian Cancer Screening Trial (PLCO):** PLCO enrolled 154,934 participants (men and women, aged between 55 and 74 years) at ten centers from 1993 to 2001 into a large, randomized, two-arm trial to determine the eﬀectiveness of screening to reduce cancer mortality. Sequential blood samples were collected from participants assigned to the screening arm. Participation was 93% at the baseline blood draw. In the observational (control) arm, buccal cells were collected via mail using the “swish-and-spit” protocol and participation rate was 65%. Details of this study have been previously described [^19^](#_ENREF_19)^,^ [^20^](#_ENREF_20) and are available online (http://dcp.cancer.gov/plco).

The Set 1 scan included a subset of 577 colon cancer cases self-reported as being non-Hispanic White with available DNA samples, questionnaire data, and appropriate consent for ancillary epidemiologic studies. Cases were excluded if they had a history of inﬂammatory bowel disease, polyps, polyposis syndrome or cancer (excluding basal or squamous cell skin cancer). Controls come from the Cancer Genetic Markers of Susceptibility (CGEMS) prostate cancer scan [^21^](#_ENREF_21) (all male) and the GWAS of Lung Cancer and Smoking [^22^](#_ENREF_22) (enriched for smokers) along with an additional 92 non-Hispanic White female controls. For the Set 2 scan, cases were colorectal cancers from both arms of the trial, which were not already included in Set 1. Samples were excluded if participants did not sign appropriate consents, if DNA was unavailable, if baseline questionnaire data with follow-up were unavailable, if they had a history of colon cancer prior to the trial, if they had a rare cancer, and if they were already in colon GWAS, or if they were a control in the prostate or lung populations. Controls were frequency matched 1:1 to cases without replacement, and cases were not eligible to be controls. Matching criteria were age at enrollment (two year blocks), enrollment date (two year blocks), sex, race/ethnicity, trial arm, and study year of diagnosis (i.e. controls must be cancer free into the case’s year of diagnosis).

**Postmenopausal Hormones Supplementary Study to the Colon Cancer Family Registry (PMH-CCFR)** ^[23](#_ENREF_23)^: Eligible case patients included all female residents, ages 50 to 74 years, residing in the 13 counties in Washington State reporting to the Cancer Surveillance SEER program, who were newly diagnosed with invasive colorectal adenocarcinoma (ICD-O C18.0, C18.2-.9, C19.9, C20.0-.9) between October 1998 and February 2002. Eligibility for all individuals was limited to those who were English-speaking with available telephone numbers, in which they could be contacted. On average, cases were identified within four months of diagnosis. The overall response proportion of eligible cases identified was 73%. Community-based controls were randomly selected according to age distribution (in 5-year age intervals) of the eligible cases by using lists of licensed drivers from the Washington State Department of Licensing for individuals, ages 50 to 64 years, and rosters from the Health Care Financing Administration (now the Centers for Medicare and Medicaid) for individuals older than 64 years. The overall response proportion of eligible controls was 66%. In GECCO, samples with sufficient DNA extracted from blood were genotyped. Only participants that were not part of the CCFR Seattle site were included in the sample set.

**VITamins And Lifestyle (VITAL):** The VITamins And Lifestyle (VITAL) cohort comprises of 77,721 Washington State men and women aged 50 to 76 years, recruited from 2000 to 2002 to investigate the association of supplement use and lifestyle factors with cancer risk. Subjects were recruited by mail, from October 2000 to December 2002, using names purchased from a commercial mailing list. All subjects completed a 24 page questionnaire and buccal-cell specimens for DNA were self-collected by 70% of the participants. Subjects are followed for cancer by linkage to the western Washington SEER cancer registry and are censored when they move out of the area covered by the registry or at time of death. Details of this study have been previously described. [^24^](#_ENREF_24) In Genetics and Epidemiology of Colorectal Cancer Consortium (GECCO), a nested case-control set was genotyped. Samples included colorectal cancer cases with DNA, excluding subjects with colorectal cancer before baseline, in situ cases, (large cell) neuroendocrine carcinoma, squamous cell carcinoma, carcinoid tumor, Goblet cell carcinoid, any type of lymphoma, including non-Hodgkin, Mantle cell, large B-cell, or follicular lymphoma. One control was randomly selected per case among all controls whose follow-up time were greater than the follow-up time of the case until diagnosis and who were matched on age at enrollment (within one year), enrollment date (within one year), sex, and race/ethnicity.

**Women’s Health Initiative (WHI):** WHI is a long-term health study of 161,808 post-menopausal women aged 50 to 79 years recruited from 1993 to 1998 at 40 clinical centers throughout the U.S. WHI comprises a Clinical Trial (CT) arm, an Observational Study (OS) arm, and several extension studies. The details of WHI have been previously described [^25^](#_ENREF_25)^,^ [^26^](#_ENREF_26) and are available online (https://cleo.whi.org/SitePages/Home.aspx). In GECCO, Set 1 cases were selected from the September 12, 2005 database and were comprised of centrally adjudicated colon cancer cases from the Observational Study (OS) who self-reported as White. Controls were ﬁrst selected among controls previously genotyped as part of a Hip Fracture GWAS conducted within the WHI OS and matched to cases on age (within three years) enrollment date (within 365 days), hysterectomy status, and prevalent conditions at baseline. For 37 cases, there was not a control match in the Hip Fracture GWAS. For these participants, we identiﬁed a matched control in the WHI OS based on same criteria. In the Set 2 scan, cases were selected from the August 2009 database and were comprised of centrally adjudicated colon and colorectal cancer cases from the OS and CT who were not genotyped in Set 1. In addition, case and control participants were subject to the following exclusion criteria: a prior history of colorectal cancer at baseline, IRB approval not available for data submission into dbGaP, and not suﬃcient DNA available. Matching criteria included age (within years), race/ethnicity, WHI date (within three years), WHI Calcium and Vitamin D study date (within three years), and randomization arms (OS ﬂag, hormone therapy assignments, dietary modiﬁcation assignments, calcium/vitamin D assignments). In addition, they were matched on the four regions of randomization centers. Each case was matched with one control (1:1) that exactly met the matching criteria. Control selection was done in a time-forward manner, selecting one control for each case ﬁrst from the risk set at the time of the case’s diagnosis. The matching algorithm was allowed to select the closest match based on a criterion to minimize an overall distance measure. [^27^](#_ENREF_27) Each matching factor was given the same weight. Additional available controls that were genotyped as part of the Hip Fracture GWAS were included to improve power.

**Colon Cancer Family Registry (CCFR)**:

**Colon Cancer Family Registry (CCFR)**: The CCFR is an NCI-supported consortium consisting of six centers dedicated to the establishment of a comprehensive collaborative infrastructure for interdisciplinary studies in the genetic epidemiology of colorectal cancer ^[28](#_ENREF_28)^. The CCFR includes data from approximately 42,500 total subjects (10,500 probands, and 32,000 unaffected and affected relatives and unrelated controls). Cases and controls, age 20 to 74 years, were recruited at the six participating centers beginning in 1998. CCFR implemented a standardized questionnaire that is administered to all participants, and includes established and suspected risk factors for colorectal cancer, which includes questions on medical history and medication use, reproductive history (for female participants), family history, physical activity, demographics, alcohol and tobacco use, and dietary factors. For genome-wide interaction analysis the CCFR scan has been described previously (Figueiredo et al, 2011), includes population-based cases and age-matched controls from the three population-based centers: Seattle, Toronto and Australia. Cases were genetically enriched by over-sampling those with a young age at onset or positive family history. Controls were matched to cases on age and sex. All cases and controls were self-reported as White, which was confirmed with genotype data.

**CORECT**

**Colocare Consortium.** The ColoCare Study (clinical trials # NCT02328677) is a prospective cohort study of newly-diagnosed colorectal cancer (CRC) patients. The ColoCare Consortium is a multicenter initiative establishing an international cohort of colorectal cancer (CRC) patients for interdisciplinary studies of CRC prognosis and outcomes with sites at the Fred Hutchinson Cancer Research Center, Seattle (Washington, USA), H. Lee Moffitt Cancer Center and Research Institute, Tampa (Florida, USA), the University Hospital Heidelberg (Germany), and the Huntsman Cancer Institute (Utah, USA). The ColoCare Study investigates clinical outcomes, including disease-free and overall survival, predictors of cancer recurrence, health-related quality-of-life, and treatment toxicities. In addition, cross-sectional analyses of biomarkers and/or health behaviors are undertaken. Patients are recruited at baseline (time of first diagnosis) and followed for up to 5 years at regular timepoints (3 months (m), 6m, 12m, 24m, 36m, 48m, 60m). The cohort includes a comprehensive collection of specimens and data.
Patients included in the CORECT project were recruited at the following ColoCare sites: Fred Hutchinson Cancer Research Center (FHCRC) and the German Cancer Research Center (DKFZ, Heidelberg, HBG). CRC patients were recruited at the ColoCare Consortium sites when consulting with a colorectal surgeon or their staff as soon as possible after their diagnosis. Inclusion criteria for the ColoCare cohort are: (1) age 18-89 years, (2) newly-diagnosed CC (stages I-III), (3) English (FHCRC, Moffitt) or German (DKFZ) speaking, and (4) mentally/physically able to consent and participate. Pregnant women and prisoners are excluded. All activities including patient identification and recruitment, administration of health behavior questionnaires, specimen collection, medical record abstraction, biospecimen and data analysis are conducted according to IRB-approved protocols. Procedures and protocols for ColoCare FHCRC are currently approved under FHCRC IRB File 6407 and ColoCare Heidelberg (HBG) IRB approval has also been obtained (University of Heidelberg, 3/10/2010).

**Colon Cancer Family Registry (CCFR).** Cohort description is provided above under CCFR. Additional participants from the CCFR consortium were included in the CORECT analysis.

**American Cancer Society Cancer Prevention Study II (CPS-II).** As described previously, CPS-II is a cohort study started by the American Cancer Society in 1982 to investigate the relationship between dietary, lifestyle and other etiologic factors and cancer mortality ^[29](#_ENREF_29)^. Approximately 1.2 million men and women were enrolled in the study from 50 states in the U.S. In 1992, a subset of these participants (N~184,000) were enrolled in the CPS-II Nutrition Cohort to examine the relationship between dietary and other exposures and cancer incidence. Blood samples were drawn from approximately 39,376 members of the Nutrition Cohort from 1998 to 2001, and buccal cells were collected from 69,467 additional members from 2001 to 2002. Cancer cases are identified by self-report through biennial follow-up questionnaires or through linkage with the National Death Index, followed by verification through medical records or linkage to state cancer registries. A total of 548 men and women diagnosed with colon or rectal cancer after providing a blood or buccal cell sample were genotyped for this study. Population-based control participants genotyped for this study included 538 men and women from the CPS-II Nutrition Cohort, individually matched to a case on sex, race/ethnicity, date of birth, date of sample collection, and DNA source (blood or buccal cell).

**Esther II/VERDI ^[30](#_ENREF_30), [31](#_ENREF_31)^*.*** In the ESTHER/VERDI study, patients diagnosed with various forms of cancer at ages 50-75, including patients with colorectal cancer (n=420), were recruited statewide in Saarland, Germany between 1996-1998 and 2001-2003. Controls, who were frequency matched by sex and age, were randomly drawn from women and men who were recruited for a statewide cohort study in Saarland, Germany when undergoing a health check-up with their general practitioners in 20002002 (n=437). Blood samples were drawn by the treating physicians who also provided medical data from their records. Risk factor information was collected by self-administered standardized questionnaires. The analytic dataset from the ESTHER/VERDI study included in the CORECT PHASE 2 GWAS consisted of 420 CRC cases and 437 controls.

**Kentucky.** The Kentucky Case-Control study was initiated in July 2003 through the University of Kentucky Cancer Center. A Supporting Information-based reporting system implemented by the Kentucky Cancer Registry in 2003 has facilitated rapid report of cases statewide, with approximately 76.8% of all cases reported to the registry within 6 months of diagnosis. Cases (>21 years) diagnosed with histologically confirmed colon cancer and entered into the registry within 6 months of their diagnoses are invited to join the study. Population-based unrelated controls are recruited through random digit dialing and are frequency matched to the cases by age (±5 years), gender, and race. Excluded from the study are those individuals who have been diagnosed with colon cancer because of known hereditary forms of colon cancer or polyposis such as familial adenomatous polyposis (FAP), hereditary non-polyposis colorectal cancer (HNPCC), Peutz-Jeghers, and Cowden disease. Currently there are more than 1,040 incident population-based cases of colorectal cancer and 1,750 population-based controls fully recruited, with comprehensive epidemiologic data, pathology data, and DNA from cases and controls

**Kiel (PopGen Biobank)** <http://f1000.com/work/citation?ids=2818119&pre=&suf=&sa=0> [^32^](#_ENREF_32). All samples used in the present study were collected through the PopGen biobank ^[19](#_ENREF_19)^. The CRC cases were members of a patient cohort from the Kiel area, described in detail elsewhere ^[21](#_ENREF_21)^. Briefly, CRC patients who had been diagnosed or operated between 2002 and 2005 were identified through the cancer registry of Schleswig-Holstein or one of 25 surgical departments in Northern Germany, and were contacted by mail between August 2004 and December 2006. A total of 2,715 patients agreed to participate (response rate: 40%). All cases eventually included in the study had histologically proven CRC, a primary CRC diagnosis, and no previous cancer. The PopGen control samples ^[33](#_ENREF_33)^ were ascertained through the local population registry of Kiel and were contacted by mail between June 2005 and February 2006. Additional control individuals were recruited by way of convenience sampling from local blood donors. Blood donors were deemed eligible for inclusion if they were healthy non-first time donors and C18 years of age. In total, data on 1,317 control individuals were available for study, including 747 PopGen population controls and 570 blood donors. Venous EDTA blood samples were collected at baseline from both cases and controls, either at the PopGen facility or by local general practitioners. Genomic DNA (600–1,000 lg) was extracted by standard methods, using the Blood Gigakit (Invitek, Berlin, Germany), and stored under quality-controlled conditions at -20°C. At the time of enrollment, all study participants completed a baseline questionnaire or interview on their personal and family history of disease (including cancer) as well as on their height, weight and lifestyle factors (such as smoking and alcohol consumption). In addition, 2,067 of the CRC cases (76%) completed a follow-up questionnaire between August 2009 and April 2010 requesting information on their body weight 1 year before CRC was diagnosed. If the patients indicated presence of a first-degree relative with CRC, they were classified as having a ‘positive family history of CRC’. Patients without a family history of CRC were classified as ‘sporadic.’

**Melbourne Collaborative Cohort Study (MCCS).** The MCCS includes both men and women volunteers, aged 40-69 and recruited from the Melbourne metropolitan area in the early 1990s. In order to recruit a sample with an increased range of dietary exposures, it was decided to deliberately enrich the cohort (25%) with migrants to Melbourne from Italy and Greece. The baseline questionnaires included questions on personal medical history and family history of common diseases. A food frequency questionnaire specially developed for the MCCS was also administered. Other important environmental variables were also accounted for. Blood samples were collected from all participants and stored as lymphocytes, buffy coats and dried blood blots. . All participants underwent an anthropmetric assessment at which direct measures of weight, height, waist and hips circumferences and bioimpedance were taken according to standard procedures by trained personnel. A total of 16962 men and 24286 women aged between 40 and 69 years were recruited into the cohort between 1990 and 1994 ^[34](#_ENREF_34)^. Cancer cases are ascertained by regular record linkage to the Victorian Cancer Registry and, for participants who have moved out of Victoria, by annual record linkage to the Australian Cancer Database and to the National Death Index at the Australian Institute of Health and Welfare in Canberra.

**MEC.** Cohort description is provided above under GECCO. Additional participants from this study were included for CORECT.

**Molecular Epidemiology of Colorectal Cancer (MECC) Study** ^[35](#_ENREF_35)^. The Molecular Epidemiology of Colorectal Cancer Study (MECC) is a population-based case-control study of colorectal cancer (CRC). Incident, pathologically-confirmed CRC cases and controls were recruited from a specific region of northern Israel. Newly-diagnosed CRC cases beginning March 31, 1998, who agreed to participate were interviewed, gave a venous blood sample, and provided permission for tumor tissue retrieval. Written, informed consent was obtained according to Institutional Review Board-approved protocols at Carmel Medical Center in Haifa and the University of Southern California (HS-12-00324, HS-12-00672, and HS-08-00378). Germline DNA was extracted from whole blood for genotyping. The analytic dataset from the MECC study genotyped on the OncoArray and included in the CORECT Phase 2 European GWAS consisted of 3,591 cases of pathologically-confirmed adenocarcinoma and 2,848 controls. In addition, previously genotyped cases and controls were included in the Phase 1 GWAS: these consisted of 484 cases and 498 controls genotyped on the Illumina Omni 2.5 array, and 1,120 cases and 820 controls were genotyped on the Affymetrix Axiom CORECT Set array. Thus, the total number of cases and controls from the MECC study included in Phases 1 and 2 (after quality control for genotyping) was 5,195 cases and 4,166 controls.

**Memorial Sloan Kettering (MSKCC).** The Memorial Sloan Kettering (MSK) cohort consisted of 126 individuals of Ashkenazi Jewish descent with a diagnosis of colorectal cancer and no known germline mutations in colon cancer predisposition genes. Eligible patients were ascertained between 2001–2013 under three existing MSK IRB-approved protocols allowing for tumor/germline biospecimen collection and germline analysis for cancer susceptibility. Two of the protocols specifically focused on ascertainment of patients with either early-onset (age ≤ 50 at diagnosis) colorectal cancer or familial colorectal cancer with no identifiable germline mutations, while the third study included colorectal cancer patients irrespective of age or family cancer history. Patient data extracted from medical records included information on stage, tumor location, chemotherapy regimen received, history of medication use (HRT NSAIDs), endoscopy results, and metachronous or synchronous colorectal or other primary cancer diagnoses.

**Newfoundland Familial Colon Cancer Registry (NFCCR)** ^[36](#_ENREF_36)^. The NFCCR is a case-control study, which includes pathology confirmed CRC cases, less than 75 years of age, diagnosed between January 1, 1999 and December 31, 2003, identified from the Newfoundland Cancer Registry. The Newfoundland Cancer Registry registers all cases of invasive cancer diagnosed among residents of the province of Newfoundland and Labrador. Consenting patients received a family history questionnaire and were asked to provide a blood sample and to permit access to tumor tissue and medical records. If a patient was deceased, we sought the participation of a close relative for the purposes of obtaining the family history and for permission to access tissue blocks and medical records. Use of proxies in this way removes the bias of excluding advanced-stage cancer patients who die before they can give consent. Controls were identified by random digit dialing from the residents of the province, and matched to the cases on sex and five year age group. Controls provided a blood sample and filled out a risk factor questionnaire.

**Nurses' Health Study II (NHSII).** The Nurses' Health Study II (NHSII) is an ongoing cohort of 116,430 female registered nurses in the US, aged 25-42 years at baseline in 1989. Demographic, lifestyle and health-related information were obtained from participants at baseline and updated every 2 years using self-administered questionnaires. The follow-up rate in each cycle has been over 90% to date. Study participants who had not previously reported a diagnosis of cancer and had responded to the 1995 NHSII study questionnaire were invited to provide blood samples between 1996 and 1999. Blood samples were collected from 29,611 NHSII participants, aged 32 to 54 years at the time of blood draw. 19 Similarly, between 2004 and 2006, active study participants who had not previously provided a blood sample were invited to provide buccal samples. Swish-and-spit sample of buccal cells were received from 29,859 participants. Cases and controls selected for genotyping were nested within the subcohort of participants who provided a blood or a buccal sample. Participants with a prior history of any cancer (except non-melanoma skin cancer), ulcerative colitis, or familial polyposis syndromes were excluded. Incident cases of colorectal adenocarcinoma were ascertained first by self-report and later confirmed by reviewing medical records and pathology reports within each follow up cycle. Deaths due to colorectal cancer were identified through family or next of kin or by querying the National Death Index. 20 Controls were randomly selected among participants in the subcohort provided they were free of colorectal cancer, and matched to a corresponding case by both age (within 1 year) and sample collection date (month/year of blood or buccal sampling). Overall, 133 cases and 132 matched controls were selected for OncoArray genotyping, and 109 cases and 102 controls with ≥80% estimated European ancestry based on STRUCTURE were included in the meta-analyses.

**Spain.** The Spanish study combines data of three case-control studies. The first one, performed in University Hospital of Bellvitge, L'Hospitalet, Barcelona, recruited 304 incident, pathology confirmed, CRC cases and 293 age and sex frequency-matched hospital controls during the period 1996-1998. The control group consisted of patients without previous colorectal cancer who had been randomly selected among those admitted to the same hospital during the same period. To avoid selection bias, the criterion of inclusion in the control group was a new diagnosis. The second study, performed in the same hospital during the period 2007-2015, included a total of 324 cases and 376 population controls. The control group was recruited by inviting to participate subjects selected from the primary health care lists of the hospital’s referral area, frequency matched by age and sex. The third study was conducted in Hospital of Leon, Leon, during 2008-2013. A total of 325 incident CRC cases and 407 population controls were included. The control population was recruited by inviting to participate subjects selected from the primary health care lists, frequency matched by age and sex. Written informed consent was required from all participants. Each Hospital's ethics committees (Bellvitge and Leon) approved the protocols of the study.

**The Swedish Low-Risk Colorectal Cancer Study.** During the years 2004-2009 more than 3300 consecutive patients operated on for colorectal cancer (CRC) in 14 hospitals in and around Stockholm and Uppsala were included in the Swedish Colorectal Cancer Low-risk study, and gave informed consent and blood for genetic studies. All cases were interviewed by the same person about their family history of colorectal cancer and other malignancies. Cancer in first- and second-degree relatives and cousins was recorded, and pedigrees for the families of the index-person (the patient) were constructed. All diagnoses in family members which could have been CRC were verified using medical records or death certificates. Other diagnoses were coded as stated by the index case. All haematological malignancies were coded as one entity as well as all gynaecological cancers because of difficulties in defining the exact diagnosis. Cases with no relative diagnosed with CRC were considered sporadic. Familial CRC was defined as cases with at least one relative with CRC in the family as defined above. All patients where relatives were at increased risk because of the family history were offered genetic counselling. Sex, age and tumor location of the index-patients were recorded based on the medical records. Tumors were assigned locations in caecum, ascending colon, hepatic flexure, transverse colon, splenic flexure, descending colon, the sigmoid or rectum. All tumors underwent evaluation directly after surgery by a local pathologist. The tumours were staged both according to the AJCC classification and the TNM-system. Some cases had two or more tumours and when tumours were located within the same segment they could be classified. As controls were used samples from 2,300 blood donors from the same region and 700 spouses to CRC patients, who did not have cancer and no family history of cancer. No information except gender were available for blood donors. For the spouse’s information on gender, age, height, weight were obtained. All patients gave written informed consents in accordance with Swedish legislation and the study was approved by the Regional research ethics committee, Dnr: 02-489.

**Swedish Mammography Cohort (SMC):** The SMC was established between 1987 and 1990 when all women who were born between 1914 and 1948 and resided in the Uppsala County of central Sweden or in the adjacent Västmanland County, received an invitation by mail to participate in a mammography screening program. Included with the invitation was a six-page questionnaire that solicited information on diet and alcohol intake, weight, height, parity, age at first childbirth, family history of breast cancer, education, and marital status. A total of 66,651 women (74% response rate) returned a completed questionnaire. In 1997 a second questionnaire was mailed to all cohort members who were still living in the study area. This questionnaire updated information on diet and was extended to include data on medical history, age at menarche, history of oral contraceptive use, age at menopause, postmenopausal hormone use, and lifestyle factors such as smoking history, physical activity, and dietary supplement use. In 2005 and in 2008, a total of 16,265 participants of the SMC received by mail an Oragene® saliva kit. Complete follow-up of participants for cancer and death has been accomplished through record linkage, using the unique personal identity number assigned to each Swedish resident at birth, with the Swedish Cancer and Cause of Death Registers. Colorectal cancer cases included in CORECT were ascertained between 1987 and 2011. We constructed a case-control set that included colorectal cancer cases and randomly selected controls (matched to the cases by year of birth) who provided a saliva sample and were free of cancer during the follow-up. The Regional Ethical Review Board at Karolinska Institutet in Stockholm approved genetic studies of CRC based on the cohorts. The analytic dataset from this study included in the CORECT Phase 2 GWAS consisted of 580 CRC cases and 859 controls.

**Studies of Epidemiology and Risk Factors in Cancer Heredity (SEARCH).** The study started recruitment on March 1, 2001 and all CRC cases diagnosed between the ages of 18 and 69 since January 1, 1996 in the regions served by the Eastern Cancer Registration and Information Centre were eligible for inclusion. Recruitment continued until the end 2010. Sex and age (in 5-year age bands) frequency matched controls were identified from the registration lists of ten representative general practices across East Anglia (England). Controls were matched to cases participating in SEARCH breast, colorectal, prostate, ovarian and endometrial cancer studies. All participants completed an epidemiological questionnaire, provided a blood sample for DNA and provided written informed consent. Genotyping was carried out on all SEARCH colorectal cancer cases and controls that had provided a blood sample and returned a completed consent form. SEARCH is approved by the Cambridgeshire 4 Research Ethics Committee

**USC Norris Comprehensive Cancer Center Genetics Registry.** The USC Norris Cancer Genetics Registry is a multicenter registry established to improve clinical care and to facilitate research to elucidate the genetic basis of hereditary cancers. Patients and families at risk of cancer are recruited from the University of Southern California Norris Comprehensive Cancer Center and LAC+USC Medical Center, where risk assessment, genetic counseling and clinical management are provided to individuals and families at risk for a variety of hereditary forms of cancer. Families at risk for hereditary forms of cancer are enrolled for continual follow-up to facilitate adherence to screening and management recommendations and to promote communication about risk to other relatives. Established in January, 2013, the USC Norris Cancer Genetics Registry provides the research structure for the development of a biorepository, clinical annotation, family expansion, longitudinal follow-up for research, germline and somatic DNA and RNA analysis, and analysis for the development of biostatistical and genetic models. For the OncoArray study, DNA samples and data from consented individuals from the USC Norris Cancer Genetics Registry with a pathologically confirmed diagnosis of adenocarcinoma of the colon or rectum were included for analysis, representing 234 colorectal cancer cases, and 221 were retained after QC. No controls were included from the registry for Oncoarray analyses.

References:

1. Day FR, Thompson DJ, Helgason H, Chasman DI, Finucane H, Sulem P et al. Genomic analyses identify hundreds of variants associated with age at menarche and support a role for puberty timing in cancer risk. *Nat Genet.* 2017.

2. Day FR, Ruth KS, Thompson DJ, Lunetta KL, Pervjakova N, Chasman DI et al. Large-scale genomic analyses link reproductive aging to hypothalamic signaling, breast cancer susceptibility and BRCA1-mediated DNA repair. *Nat Genet.* 2015; **47**: 1294-1303.

3. Burgess S. Sample size and power calculations in Mendelian randomization with a single instrumental variable and a binary outcome. *International journal of epidemiology.* 2014; **43**: 922-929.

4. Locke AE, Kahali B, Berndt SI, Justice AE, Pers TH, Day FR et al. Genetic studies of body mass index yield new insights for obesity biology. *Nature.* 2015; **518**: 197-206.

5. Burgess S, Butterworth A, Thompson SG. Mendelian randomization analysis with multiple genetic variants using summarized data. *Genetic epidemiology.* 2013; **37**: 658-665.

6. Bowden J, Davey Smith G, Burgess S. Mendelian randomization with invalid instruments: effect estimation and bias detection through Egger regression. *International journal of epidemiology.* 2015; **44**: 512-525.

7. Bowden J, Davey Smith G, Haycock PC, Burgess S. Consistent Estimation in Mendelian Randomization with Some Invalid Instruments Using a Weighted Median Estimator. *Genetic epidemiology.* 2016; **40**: 304-314.

8. Yavorska OO, Burgess S. MendelianRandomization: an R package for performing Mendelian randomization analyses using summarized data. *International journal of epidemiology.* 2017.

9. Kury S, Buecher B, Robiou-du-Pont S, Scoul C, Sebille V, Colman H et al. Combinations of cytochrome P450 gene polymorphisms enhancing the risk for sporadic colorectal cancer related to red meat consumption. *Cancer epidemiology, biomarkers & prevention : a publication of the American Association for Cancer Research, cosponsored by the American Society of Preventive Oncology.* 2007; **16**: 1460-1467.

10. Le Marchand L, Hankin JH, Wilkens LR, Pierce LM, Franke A, Kolonel LN et al. Combined effects of well-done red meat, smoking, and rapid N-acetyltransferase 2 and CYP1A2 phenotypes in increasing colorectal cancer risk. *Cancer epidemiology, biomarkers & prevention : a publication of the American Association for Cancer Research, cosponsored by the American Society of Preventive Oncology.* 2001; **10**: 1259-1266.

11. Brenner H, Chang-Claude J, Seiler CM, Rickert A, Hoffmeister M. Protection from colorectal cancer after colonoscopy: a population-based, case-control study. *Annals of internal medicine.* 2011; **154**: 22-30.

12. Lilla C, Verla-Tebit E, Risch A, Jager B, Hoffmeister M, Brenner H et al. Effect of NAT1 and NAT2 genetic polymorphisms on colorectal cancer risk associated with exposure to tobacco smoke and meat consumption. *Cancer epidemiology, biomarkers & prevention : a publication of the American Association for Cancer Research, cosponsored by the American Society of Preventive Oncology.* 2006; **15**: 99-107.

13. Slattery ML, Potter J, Caan B, Edwards S, Coates A, Ma KN et al. Energy balance and colon cancer--beyond physical activity. *Cancer research.* 1997; **57**: 75-80.

14. Kolonel LN, Henderson BE, Hankin JH, Nomura AM, Wilkens LR, Pike MC et al. A multiethnic cohort in Hawaii and Los Angeles: baseline characteristics. *American journal of epidemiology.* 2000; **151**: 346-357.

15. Belanger CF, Hennekens CH, Rosner B, Speizer FE. The nurses' health study. *The American journal of nursing.* 1978; **78**: 1039-1040.

16. Cotterchio M, McKeown-Eyssen G, Sutherland H, Buchan G, Aronson M, Easson AM et al. Ontario familial colon cancer registry: methods and first-year response rates. *Chronic diseases in Canada.* 2000; **21**: 81-86.

17. Cotterchio M, Manno M, Klar N, McLaughlin J, Gallinger S. Colorectal screening is associated with reduced colorectal cancer risk: a case-control study within the population-based Ontario Familial Colorectal Cancer Registry. *Cancer causes & control : CCC.* 2005; **16**: 865-875.

18. Zanke BW, Greenwood CM, Rangrej J, Kustra R, Tenesa A, Farrington SM et al. Genome-wide association scan identifies a colorectal cancer susceptibility locus on chromosome 8q24. *Nat Genet.* 2007; **39**: 989-994.

19. Prorok PC, Andriole GL, Bresalier RS, Buys SS, Chia D, Crawford ED et al. Design of the Prostate, Lung, Colorectal and Ovarian (PLCO) Cancer Screening Trial. *Controlled clinical trials.* 2000; **21**: 273S-309S.

20. Gohagan JK, Prorok PC, Hayes RB, Kramer BS. The Prostate, Lung, Colorectal and Ovarian (PLCO) Cancer Screening Trial of the National Cancer Institute: history, organization, and status. *Controlled clinical trials.* 2000; **21**: 251S-272S.

21. Yeager M, Chatterjee N, Ciampa J, Jacobs KB, Gonzalez-Bosquet J, Hayes RB et al. Identification of a new prostate cancer susceptibility locus on chromosome 8q24. *Nat Genet.* 2009; **41**: 1055-1057.

22. Landi MT, Chatterjee N, Yu K, Goldin LR, Goldstein AM, Rotunno M et al. A Genome-wide Association Study of Lung Cancer Identifies a Region of Chromosome 5p15 Associated with Risk for Adenocarcinoma. *American journal of human genetics.* 2011; **88**: 861.

23. Newcomb PA, Zheng Y, Chia VM, Morimoto LM, Doria-Rose VP, Templeton A et al. Estrogen plus progestin use, microsatellite instability, and the risk of colorectal cancer in women. *Cancer research.* 2007; **67**: 7534-7539.

24. White E, Patterson RE, Kristal AR, Thornquist M, King I, Shattuck AL et al. VITamins And Lifestyle cohort study: study design and characteristics of supplement users. *American journal of epidemiology.* 2004; **159**: 83-93.

25. Hays J, Hunt JR, Hubbell FA, Anderson GL, Limacher M, Allen C et al. The Women's Health Initiative recruitment methods and results. *Annals of epidemiology.* 2003; **13**: S18-77.

26. Design of the Women's Health Initiative clinical trial and observational study. The Women's Health Initiative Study Group. *Controlled clinical trials.* 1998; **19**: 61-109.

27. Bergstralh EJ, Kosanke JL, Jacobsen SJ. Software for optimal matching in observational studies. *Epidemiology (Cambridge, Mass).* 1996; **7**: 331-332.

28. Newcomb PA, Baron J, Cotterchio M, Gallinger S, Grove J, Haile R et al. Colon Cancer Family Registry: an international resource for studies of the genetic epidemiology of colon cancer. *Cancer epidemiology, biomarkers & prevention : a publication of the American Association for Cancer Research, cosponsored by the American Society of Preventive Oncology.* 2007; **16**: 2331-2343.

29. Calle EE, Rodriguez C, Jacobs EJ, Almon ML, Chao A, McCullough ML et al. The American Cancer Society Cancer Prevention Study II Nutrition Cohort: rationale, study design, and baseline characteristics. *Cancer.* 2002; **94**: 2490-2501.

30. Jansen L, Herrmann A, Stegmaier C, Singer S, Brenner H, Arndt V. Health-related quality of life during the 10 years after diagnosis of colorectal cancer: a population-based study. *Journal of clinical oncology : official journal of the American Society of Clinical Oncology.* 2011; **29**: 3263-3269.

31. Breitling LP, Raum E, Muller H, Rothenbacher D, Brenner H. Synergism between smoking and alcohol consumption with respect to serum gamma-glutamyltransferase. *Hepatology (Baltimore, Md).* 2009; **49**: 802-808.

32. Siegert S, Hampe J, Schafmayer C, von Schonfels W, Egberts JH, Forsti A et al. Genome-wide investigation of gene-environment interactions in colorectal cancer. *Human genetics.* 2013; **132**: 219-231.

33. Krawczak M, Nikolaus S, von Eberstein H, Croucher PJ, El Mokhtari NE, Schreiber S. PopGen: population-based recruitment of patients and controls for the analysis of complex genotype-phenotype relationships. *Community genetics.* 2006; **9**: 55-61.

34. Giles GG, English DR. The Melbourne Collaborative Cohort Study. *IARC scientific publications.* 2002; **156**: 69-70.

35. Poynter JN, Gruber SB, Higgins PD, Almog R, Bonner JD, Rennert HS et al. Statins and the risk of colorectal cancer. *The New England journal of medicine.* 2005; **352**: 2184-2192.

36. Woods MO, Younghusband HB, Parfrey PS, Gallinger S, McLaughlin J, Dicks E et al. The genetic basis of colorectal cancer in a population-based incident cohort with a high rate of familial disease. *Gut.* 2010; **59**: 1369-1377.
